# Probability of stealth multiplets in sample-multiplexing for droplet-based single-cell analysis

**DOI:** 10.1186/s12864-025-11835-z

**Published:** 2025-07-23

**Authors:** Fumio Nakaki, James Sharpe

**Affiliations:** 1 European Molecular Biology Laboratory, EMBL Barcelona, Carrer del Dr. Aiguader, 88, PRBB Building, 08003 Barcelona, Spain; 2 Institució Catalana de Recerca i Estudis Avançats (ICREA), Passeig Lluís Companys 23, 08010 Barcelona, Spain; 3 Barcelona Collaboratorium for Modelling and Predictive Biology, Carrer de Wellington, 30, 08005 Barcelona, Spain

**Keywords:** Single-cell analysis, scRNA-seq, Sample multiplexing, Poisson distribution, Stealth multiplet

## Abstract

**Background:**

One of the technical limits of droplet-based single-cell RNA sequencing (scRNA-seq) is the presence of multiplets, i.e. droplets that capture multiple cells. Sample-multiplexing scRNA-seq (mx-scRNA-seq) enables us to evaluate large numbers of different samples or experiments simultaneously by reducing the occurrence of undetectable multiplets. However, there is still a possibility of hidden multiplets among what appear to be singlets, for which we introduce the term stealth multiplets, and their probability is yet to be quantitatively examined.

**Results:**

We developed a simple theoretical model to predict four classes of possible multiplets in mx-scRNA-seq: Homogeneous stealth, partial stealth, multilabelled, and unlabelled. We estimated the probability of each class and have found that the partial stealth multiplet, which has been previously overlooked, may impact the results of the whole dataset, particularly when the labelling process or demultiplexing is suboptimal. Also, we demonstrated their presence in real mx-scRNA-seq datasets both in oligonucleotide-barcode demultiplexing and genotype-based demultiplexing.

**Conclusion:**

Our results show the importance of optimising the labelling procedure and choosing the most suitable demultiplexing algorithm. We thus offer a theoretical basis to estimate the probability of each type of multiplet to ensure the integrity of mx-scRNA-seq.

**Supplementary Information:**

The online version contains supplementary material available at 10.1186/s12864-025-11835-z.

## Background

Recent advances in single-cell transcriptome analysis have greatly expanded the number of genes and cells that can be analysed in a single experiment, allowing the characterisation of a massive number of individual cells simultaneously, capturing a representative transcriptome landscape of a given biological system and providing us with new insights into complex biological systems [1–7]. The microfluidic-based single-cell RNA sequencing [1, 2, 6] (hereafter, we use the term “scRNA-seq” specifically for this method), with which transcripts of an individual cell are barcoded with a unique nucleotide in a droplet generated in a microfluidic device, can theoretically label as many cells as the variation of the barcode sequences allows. However, one of the critical limiting factors is the presence of multiplets, i.e. droplets having more than one cell [1, 2, 6], which threatens the integrity of scRNA-seq. Controlling the frequency of multiplets is, therefore, a critical quality control step in scRNA-seq. An in silico multiplet remover, an algorithm to detect multiplets based on their transcriptome pattern [8–11], is a widely applicable approach, though these algorithms cannot distinguish the “homotypic” multiplets that consist of cells with very similar transcriptome profiles [8–11].

In sample-multiplexing scRNA-seq (mx-scRNA-seq), each sample has a unique feature that can be subsequently distinguished by sequencing analysis, followed by sample pooling and transcript barcoding on a microfluidic device. These features include external labelling such as oligonucleotide-conjugated antibodies [12, 13], lipid-conjugated oligonucleotides [14], click chemistry [15], and nucleotide transfection [16], or inherent genetic variation (e.g. single-nucleotide polymorphisms (SNPs)) [17, 18]. This allows us not only to increase the number of samples we can handle in one barcoding reaction but also to accurately compare multiple samples by removing an inevitable technical batch effect across samples, which broadens the application of scRNA-seq [19]. Moreover, these additional sample labels effectively reveal the multiplets that appear as droplets with multiple sample labels, which, in turn, allows us to raise the ceiling of cell number per single run by mitigating the risk of undetectable multiplets [14, 20].

However, even in mx-scRNA-seq, there is still the problem that single-labelled (hereafter monolabelled) droplets are not always true singlets. For example, a multiplet comprising multiple cells with the same sample barcode cannot be detected by barcode demultiplexing [11, 14, 20, 21]. Another potential form of hidden multiplets is a combination of labelled and unlabelled cells, which has not been examined so far. Quantitative estimation and control of these false singlets—“stealth multiplets” hereafter—is essential for experimental design and data analysis in the mx-scRNA-seq. In this article, we estimate the probabilities of stealth multiplets based on a Poisson distribution model and illustrate their presence in actual oligonucleotide-based and SNP-based mx-scRNA-seq datasets.

## Methods

### Theoretical probabilities of stealth multiplets in mx-scRNA-seq

**Fig. 1:**
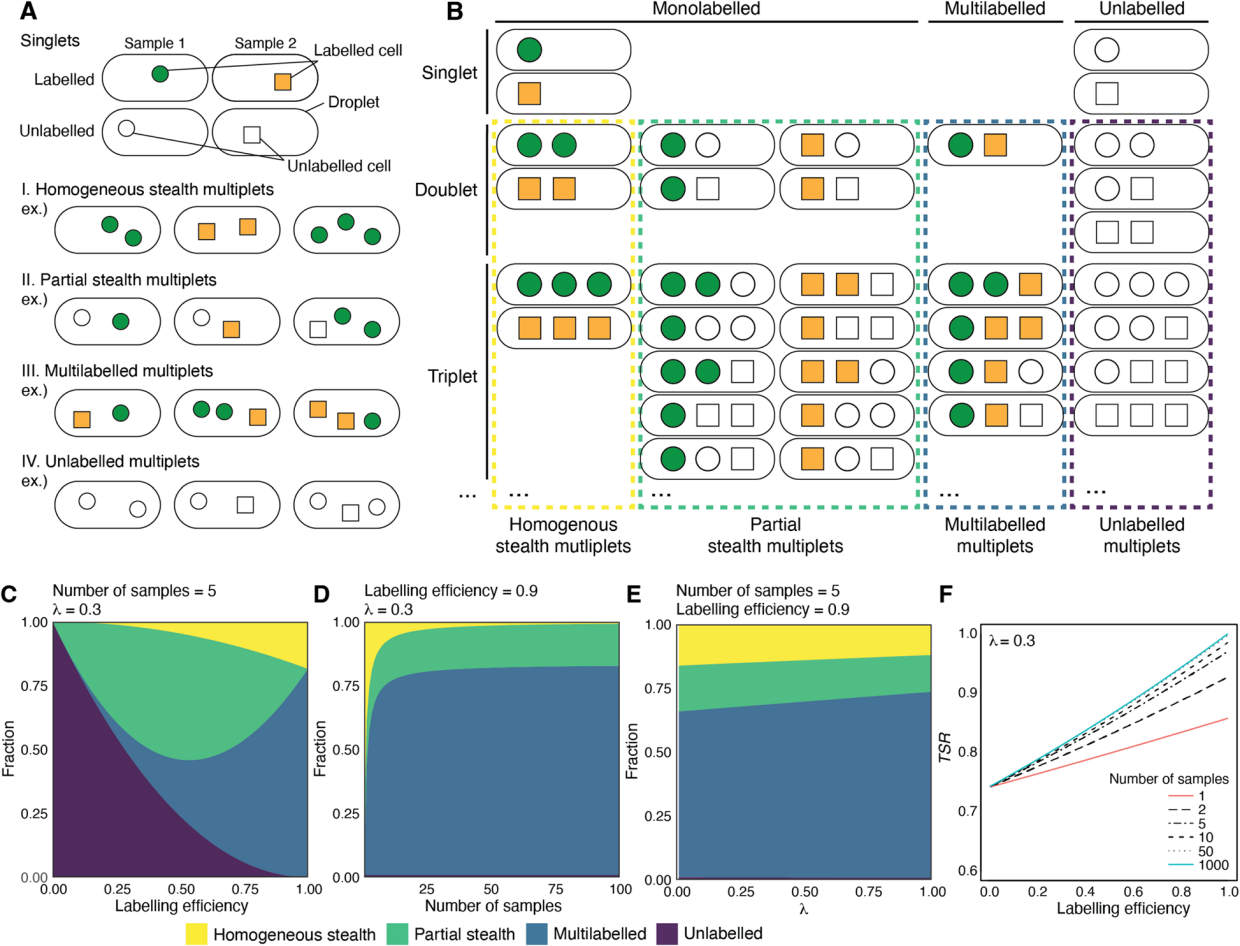
Theoretical probability of the four types of multiplets in mx-scRNA-seq and their presence in actual datasets. **A** Schematic illustration of the four types of multiplets possibly appearing in mx-scRNA-seq. Two-sample multiplex examples are shown. Labelled cells are shown in green or orange, depending on the sample labels, and unlabelled in white. The shapes represent the sample to which cells belong. In a sample multiplexing configuration, the multiplets are classified into four categories: Homogeneous stealth, partial stealth, multilabelled, and unlabelled multiplets, depending on combinations of cells in a single droplet. **B** The possible cell composition in a droplet containing at least one cell when two samples are multiplexed. The colour and shape of the cells are the same as (**A**). The probability of each category was calculated based on the combinatorics illustrated here. **C** An area chart exhibits the fraction of the four categories among multiplets in a hypothetical experiment, multiplexing five samples. $\lambda$ is a parameter of the Poisson distribution, representing the average number of cells per droplet, closely correlated with a cell loading rate in practice. We assumed all samples had an equal labelling efficiency and were pooled with an equal proportion. Proportions were plotted against labelling efficiency when $\lambda =0.3$. **D** Proportions were plotted against the number of samples when a labelling efficiency was 0.9 and $\lambda =0.3$. **E** Proportions were plotted against $\lambda$ when a labelling efficiency was 0.9 and 5 samples were multiplexed. **F***TSR* is plotted against labelling efficiency under various sample numbers. $\lambda$ is fixed at 0.3

The probabilities of each category, $p_{\textrm{HS}}$, $p_{\textrm{PS}}$, $p_{\textrm{Mu}}$, and $p_{\textrm{Un}}$ for the homogeneous stealth, partial stealth, multilabelled, and unlabelled multiplet, respectively, were estimated with

$$\begin{aligned} p_{\textrm{HS}}= & e^{-\lambda }\left( \sum \limits _{i=1}^{s}e^{r_ia_i\lambda }-\bar{a}{\lambda }-s\right) \\ p_{\textrm{PS}}= & (e^{-\bar{a}\lambda }- e^{-\lambda })\left( \sum \limits _{i=1}^{s}e^{r_ia_i\lambda }-s\right) \\ p_{\textrm{Mu}}= & 1-e^{-\bar{a}\lambda }\left( \sum \limits _{i=1}^{s}e^{r_ia_i\lambda } - s + 1 \right) \\ p_{\textrm{Un}}= & e^{-\lambda } (e^{(1-\bar{a})\lambda } - (1-\bar{a}){\lambda } -1) \\ \end{aligned}$$

where *s* is the number of samples, $a_i$ is the labelling efficiency of sample *i* ($1\le i \le s$), comprising a fraction $r_i\ (1\le i \le s,\ \sum _{i=1}^{s}r_i=1)$ of the pooled population. $\bar{a}$ is the overall average of labelling efficiencies ($\bar{a}=\sum _{i=1}^{s}r_ia_i$), and $\lambda$ is the expected value of the Poisson distribution (See Supplementary Information Section 1 and 3 for the details). Note that $p_{\textrm{Un}}$ depends on $\bar{a}$ because unlabelled multiplets are generated across samples, and the origin of cells consisting of unlabelled droplets cannot be distinguished. Also, $p_{\textrm{PS}}$ is expressed both by the overall average $\bar{a}$ and by the individual labelling efficiency, which supports the idea that the partial stealth multiplets of each sample are formed with unlabelled cell(s) from other samples. To better understand the characteristics of these probabilities, we shall assume that all samples had an equal labelling efficiency and were pooled with an equal proportion ($r_ia_i=\frac{\bar{a}}{s}$ for a given *i*) for simplicity, and in this case, $p_{\textrm{HS}}$, $p_{\textrm{PS}}$, and $p_{\textrm{Mu}}$ are

$$\begin{aligned} p_{\textrm{HS}}= & se^{-\lambda }(e^{\frac{\bar{a}\lambda }{s}}-1)-\bar{a}{\lambda }e^{-\lambda }\\ p_{\textrm{PS}}= & s(e^{-\bar{a}\lambda }-e^{-\lambda })(e^{\frac{\bar{a}\lambda }{s}}-1) \\ p_{\textrm{Mu}}= & 1-e^{-\bar{a}\lambda } \left( s(e^{\frac{\bar{a}\lambda }{s}} - 1) +1 \right) \end{aligned}$$

with which we plotted the theoretical proportion of the four categories in Fig. 1C-E. Deviation from this assumption is discussed in Supplementary Information Section 4

We defined the true singlet ratio (*TSR*) for the multiplex experiment. When *s* samples are multiplexed, the overall *TSR* is given by

$$\begin{aligned} TSR =\frac{P(1)\sum _{i=1}^{s}r_ia_i}{P(1)\sum _{i=1}^{s}r_ia_i + p_{\textrm{HS}} +p_{\textrm{PS}}} =\frac{\bar{a}{\lambda }e^{-\lambda (1-\bar{a})}}{\sum _{i=1}^{s}e^{r_ia_i\lambda }-s} \end{aligned}$$

Under the condition $r_ia_i=\frac{\bar{a}}{s}$ for every *i*, the *TSR* is

$$\begin{aligned} TSR =\frac{\bar{a}{\lambda }e^{-\lambda (1-\bar{a})}}{s (e^{\frac{\bar{a}\lambda }{s}}-1)} \end{aligned}$$

All graphical illustration was done with R (version 4.1.2) [22], and linear regression was performed with the stats::lm function.

### Mice

All mice were bred at the PRBB Animal Facility, which is accredited by the International Association for Assessment and Accreditation of Laboratory Animal Care (AAALAC), and handling and care were done in accordance with the AAALAC criteria. All procedures in this study were approved by the EMBL Institutional Animal Care and Use Committee (IACUC), and mouse embryos were collected by following the American Veterinary Medical Association (AVMA) guidelines. The B6.129-*Sox9*^*tm1Haak*^ (Sox9-EGFP) mice (MGI:3844977) [23] were maintained by backcrossing to C57BL/6J mice.

### scRNA-seq

#### Sample preparation

A hindlimb bud’s distal region corresponding to the future autopod was obtained from a Sox9-EGFP mouse embryo at E11.5. Cell attachments were loosened by incubating with TrypLE Express (Thermo Fisher Scientific) at 37$^{\circ }$C for 15 minutes, and the ectoderm was removed under the stereoscope. The isolated mesenchyme clump was put in a Cell Prep Buffer (Ca^2+^, Mg^2+^-free phosphate-buffered saline (PBS) with 0.1% poly(vinyl alcohol) (PVA) and 1 mM ethylenediaminetetraacetic acid (EDTA)) and dissociated by gentle pipetting. NIH3T3 cells were cultured with high-glucose Dulbecco’s Modified Eagle Medium (DMEM) (Thermo Fisher Scientific) supplemented with 10% Fetal Bovine Serum (FBS), L-Glutamine, and Penicillin/Streptomycin. The transgenic T-p2a-H2B-eGFP and CAG::H2B-mKO2 mouse embryonic stem (ES) cells [24] derived from G4 ES cells [25] were cultured with DMEM supplemented with 10% FBS and leukemia inhibitory factor (LIF) as described [26]. Both cultures were dissociated with TrypLE Express at 37$^{\circ }$C for 3 minutes after multiple washes with PBS to remove serum. The reaction was stopped by adding four volumes of a Wash Buffer (1:1 mixture of DMEM and Ham’s F12 medium (Thermo Fisher Scientific) with 0.1% bovine serum albumin (BSA)) to TrypLE Express, and cells were dissociated entirely by gentle pipetting. After pelleting the cells by centrifugation, they were resuspended in the designated buffer for sample labelling as described below.

#### 3’ CellPlex

**Fig. 2:**
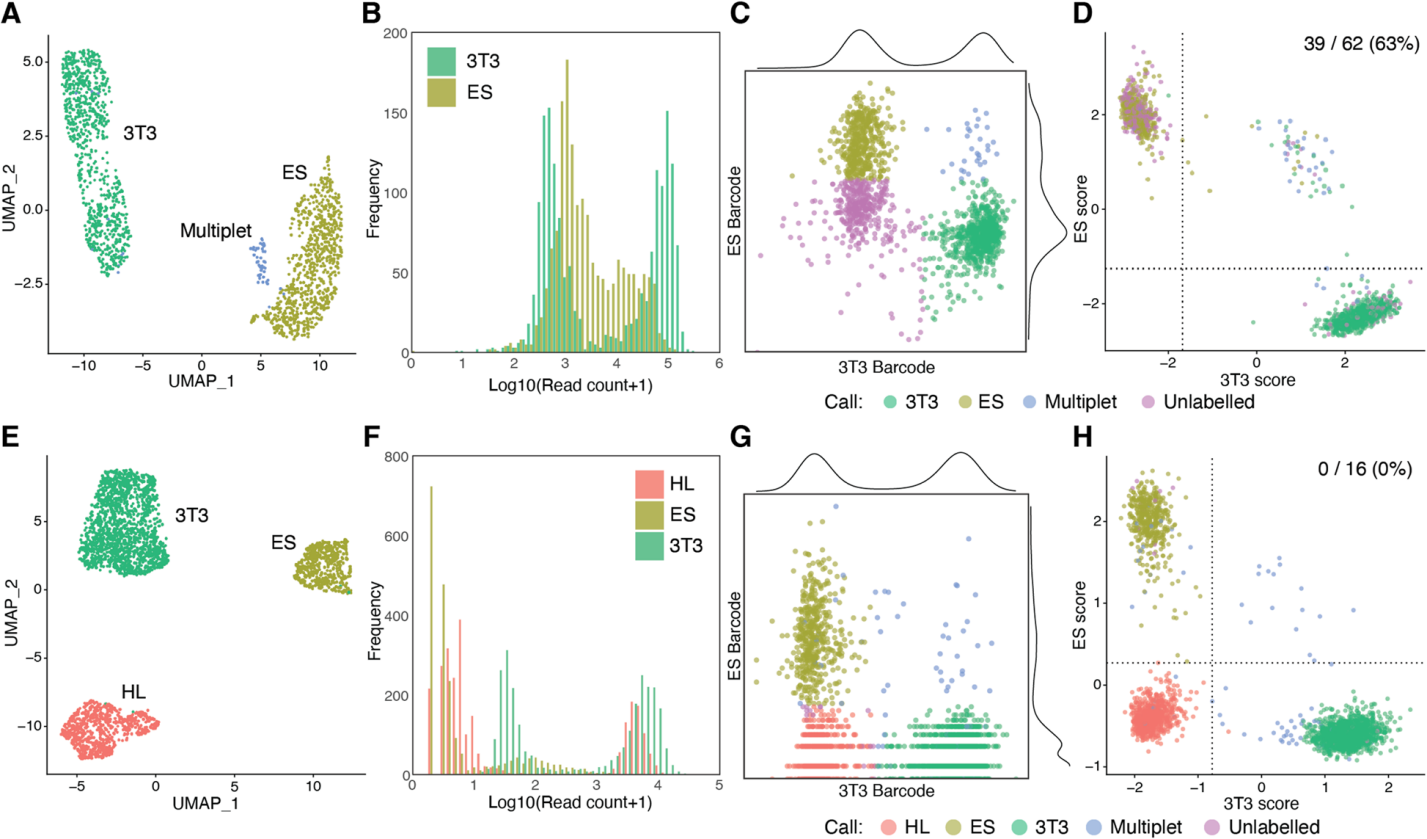
The presence of partial stealth multiplets in actual mx-scRNA-seq datasets. **A** A dimension reduction plot by UMAP on the transcriptome of mx-scRNA-seq multiplexing 3T3 and ES cells. **B** A histogram of log-transformed barcode read count per cell, grouped by each sample barcode. **C** A log-transformed scatter plot of the barcode reads, colour-coded by the demultiplexing results by GMM-demux. Each barcode density plot (similar to the histogram in (**B**)) is also shown along each axis. **D** A scatter plot of the 3T3 and ES transcriptome scores (Supplementary Fig. 2D). The cells in the right upper quadrant were regarded as multiplets, defined as double-positive cells for both cell type markers. The number of cells classified as monolabelled droplets among the multiplets (i.e. partial stealth multiplets), the total number of multiplets, and their percentage are shown at the top right. **E** A UMAP plot of the transcriptome of mx-scRNA-seq multiplexing HL, ES, and 3T3. Unlike the two-sample data in (**A**), multiplets belonged to one of the three cell lineage clusters. **F** A histogram of barcode read count per cell grouped by each sample barcode. **G** A log-transformed scatter plot of the 3T3 and ES barcode reads, colour-coded by the demultiplexing results by GMM-demux. Each barcode density plot (similar to the histogram in (**F**)) is also shown along each axis. **H** A scatter plot of the 3T3 and ES transcriptome scores (Supplementary Fig. 4A). The numbers shown at the top right are similar to (**D**)

For the two-sample-multiplexed dataset (Fig. 2A-D, Supplementary Fig. 2), sample labelling was done with 3’ CellPlex Kit Set A (10x Genomics), according to the manufacturer’s protocol. Briefly, harvested NIH3T3 cells and ES cells were resuspended in PBS with BSA (PBS-BSA) (0.04%) and filtered through a 35-${\upmu }$m cell strainer. After counting cells, 1.9$\times$10^6^ of NIH3T3 cells and 1.6$\times$10^5^ of ES cells were collected by centrifugation and resuspended in 100 ${\upmu }$L of Cell Multiplexing Oligo (CMO) 301 and CMO 302, respectively. After 5 minutes of incubation on ice, samples were washed with PBS-BSA (1%) three times and resuspended in PBS-BSA (1%), followed by cell counting. These samples were pooled so that the pooled sample contained equal numbers of cells from each cell type. The pooled samples were again filtered with a 35-${\upmu }$m cell strainer before loading onto the microfluidic chip. Cell encapsulation and barcoding were performed with 3’ Next GEM Single Cell 3’ Kit v3.1 (10x Genomics) and a Chromium Single Cell Chip G Kit (10x Genomics) by the Chromium Controller (10x Genomics, firmware version 4.00) according to the manufacturer’s protocol. The 3’-enriched cDNA and Feature Barcodes libraries were prepared with 3’ Next GEM Single Cell 3’ Kit v3.1 (10x Genomics) and Feature Barcode Kit (10x Genomics) following the manufacturer’s instructions. These libraries were sequenced with NextSeq2000 (illumina). We read 10 base pairs (bp) for i7 and i5 indices, 28 bp for 10x barcodes and UMIs, and 90 bp for both fragmented cDNA and Feature Barcodes.

#### MULTI-seq

The three-sample-multiplexed dataset (Fig. 2E-H, Supplementary Figs. 3 and 4) was generated with MULTI-seq [14]. The hindlimb bud (HL), ES, and NIH3T3 cells were resuspended in a Cell Prep Buffer and filtered through a 35-${\upmu }$m cell strainer cap. After counting cells, 1$\times$ 10^5^ cells were pelleted and resuspended in 180 ${\upmu }$L of Cell Prep Buffer. These cell samples were labelled with MULTI-seq barcode oligonucleotides as described [14]. Briefly, a 1:1 mixture of the cholesterol-conjugated Anchor-oligonucleotide (Anchor CMO, synthesised by Integrated DNA Technologies) and the Barcode oligonucleotide with a distinct barcode sequence for each sample (final concentration, 0.2 ${\upmu }$M) was added to each cell sample and incubated on ice for 5 minutes. Next, the same concentration of Co-Anchor CMO (synthesised by Integrated DNA Technologies) was added and incubated for another 5 minutes. After labelling, the labelled cells were washed with PBS-BSA (1%) three times, resuspended in 100 ${\upmu }$L of PBS-BSA (1%), and pooled. The pooled sample was filtered through a 35-${\upmu }$m cell strainer and counted again before loading onto the microfluidic chip described below. The sample-barcode sequences for the HL, ES, and NIH3T3 cells were “CATAGAGC”, “TCCTCGAA”, and “GTGTACCT”, respectively.

The pooled single-cell suspension was encapsulated with Chromium Single Cell 3’ GEM, Library & Gel Bead Kit v2 (10x Genomics) and a Chromium Single Cell Chip A Kit (10x Genomics) by the Chromium Controller (10x Genomics, firmware version 4.00) and transcripts and MULTI-seq barcode oligos were captured and reverse-transcribed as described in the manufacturer’s instructions. We targeted 4,000 cells to sequence. The 3’-enriched cDNA library construction was performed with Chromium Single Cell 3’ GEM, Library & Gel Bead Kit v2 (10x Genomics) according to the manufacturer’s instructions. The MULTI-seq barcode oligos were fractionated after the cDNA amplification, amplified and indexed by PCR with KAPA HiFi HotStart ReadyMix (Roche) for sequencing. Both cDNA and MULTI-seq-barcode libraries were sequenced with NextSeq500 (Illumina). We read 8 base pairs (bp) for TruSeq indices, 26 bp for 10x barcodes and unique molecular identifiers (UMIs), and 58 bp for fragmented cDNA or MULTI-seq barcodes.

### Data analysis

#### Quality control of scRNA-seq

The sequencing reads were aligned and mapped on the reference mouse genome (mm10) and counted for each feature to build a feature-cell matrix with CellRanger count (version 6.0.1, 10x Genomics) for MULTI-seq or CellRanger multi (version 6.1.1, 10x Genomics) for 3’CellPlex. The raw feature-cell matrix was used for 3’CellPlex, which was further screened with the R package DropletUtils (version 1.14.2) [27] for cell-droplet (a droplet containing at least one cell) candidates. For MULTI-seq, the filtered feature-cell matrix from the CellRanger was used. These feature-cell matrices were further screened for quality control with the number of genes detected and mitochondrial gene reads (Supplementary Fig. 9). Next, gene expression levels were normalised with the scater (version 1.22.0) [28] and scran (version 1.22.1) [29] packages in R (version 4.1.2) [22].

#### Clustering and visualisation

The normalised data were further analysed to remove a technical variance by a Poisson noise model, and biological variance for each gene was estimated with the scran package (version 1.22.1) [29]. Highly variable genes (HVGs) for dimension reduction were defined as the genes with a biological variance >0.1 (1,714 genes) and >0.3 (2,461 genes) for the three-sample and the two-sample dataset, respectively. Before dimension reduction, the transcriptome data were standardised with the ScaleData function in the Seurat package (version 4.3.0, hereafter Seurat) [30] in R. Converting the SingleCellExperiment class object [31] into the Seurat object was done by the as.Seurat function in Seurat. Principal component analysis (PCA) was performed on the selected HVGs, followed by Uniform Manifold Approximation and Projection (UMAP) using the first 20 principal components (PCs) from the PCA, as implemented in Seurat with default settings. Clustering was done with the FindCluster function in Seurat with the default original Louvain algorithm, with the resolution 0.1 and 0.01 for the three-sample and the two-sample datasets, respectively.

#### Sample Barcode demultiplexing

For MULTI-seq, counting sample barcodes and building a cell-barcode read count matrix were done with the MULTIseq.preProcess and MULTIseq.align functions of the deMULTIplex package (version 1.0.2) in R (version 4.1.2). For the 3’CellPlex, the Feature Barcode tag was counted with CellRanger multi (version 6.1.1, 10x Genomics). We examined three different demultiplexing algorithms for the Two- and Three-sample datasets:

deMULTIplex (version 1.0.2) [14] in R (version 4.1.2): We applied two rounds of the findThresh and classifyCells functions, followed by removing the label-negative cells at the end of each round. The assignment results after two rounds were adopted.

Seurat::HTODemux (version 4.3.0) [30] in R (version 4.1.2): First, each barcode count was normalised with centred log-ratio (CLR) transformation with the NormalizeData(normalization.method = “CLR”) function. Then, cells were classified with the HTODemux(kfunc = “clara”, positive.quantile = 0.99) function based on the normalised barcode matrix.

GMM-demux (version 0.2.1.3) [21]: Demultiplexing was done with the default confidential score threshold, -t 0.8, and designating the expected number of cells by -u 2697 and -u 1645 options for the three-sample and the two-sample dataset, respectively.

#### Detecting the partial stealth multiplet

The marker genes for each transcriptome cluster were defined using the FindMarkers function in Seurat. We adopted the top 25 differentially expressed genes as marker genes and calculated each marker score with the AddModuleScore function in Seurat. To distinguish the high score population from others, we fitted two Gaussian curves to the score distribution and cut-off at the threshold as $p=0.0001$, which is estimated from the average of the lower curve with the twoGaussiansNull(p.adj.method = “BY”, max.adj.p = 0.0001) function of the Ringo package (version 1.58.0) [32] in R. Cell-droplets with the transcriptome scores above the thresholds for more than one lineage were defined as multiplets. Among them, the ones classified as monolabelled cell-droplets by CMO labelling were judged as partial stealth multiplets.

### SNP-based demultiplexing

#### CITE-seq PBMC dataset

The original read files were obtained at Gene Expression Omnibus (Accession No. GSE108313). The read files were aligned and mapped with CellRanger (version 6.1.1, 10x Genomics) to the human genome reference (hg19). The filtered cell barcodes were extracted from the original feature count matrix file (Available at https://satijalab.org/seurat/articles/hashing_vignette). The original Hashtag oligonucleotide (HTO) count matrix (Available at the same website above) was demultiplexed with the R package, deMULTIplex2 (version 1.0.1) [33]. An estimated average labelling efficiency was 99.5%; therefore, we speculated the proportion of the partial stealth multiplet was <0.2% (Supplementary Information Section 5 for detailed estimation procedures). We considered this negligible and decided to regard this assignment as a reference “ground truth” data.

SNP-based demultiplexing was performed with demuxalot (version 0.4.1) [34], using the re-mapped bam file above and a donor genotype VCF file [20] (A kind gift from Rahul Satija), both having hg19 coordinates, with a refined SNP mode by the Demultiplexer.learn_genotypes(doublet_prior=0.25) function. In total, 550,771 SNPs were parsed from the reference file. The cut-off value of the posterior probability was 0.5. To visualise the CMO count matrix, the CMO counts were standardised with the ScaleData function of Seurat, followed by PCA. UMAP was performed with all 8 PCs with the Seurat RunUMAP(min.dist=0.75, n.neighbors=30) function. For the subsequent in silico doublet remover analysis, we used the original feature count matrix file rather than our remapped results. We normalised and standardised it according to the Seurat vignette above.

#### Total-Seq NSCLC dataset

The 40k Mixture of NSCLC DTCs from 7 donors, 3’ HT v3.1 dataset (10x Genomics) was obtained at https://www.10xgenomics.com/datasets/40-k-mixture-of-nsclc-dt-cs-from-7-donors-3-ht-v-3-1-3-1-high-6-1-0, which contained the Gene Expression - Feature cell matrix HDF5 (raw) file (40k_NSCLC_DTC_3p_HT_nextgem_Multiplex_count_raw_feature_bc_matrix.h5), aligned bam files of each donor and unassigned reads, and the Multiplexing Analysis - Number of Calls per Cell (csv) file (40k_NSCLC_DTC_3p_HT_nextgem_Multiplex_multiplexing_analysis_tag_calls_per_cell.csv) in which the 10x Genomics pre-filtered and listed cell-droplet barcodes in this dataset. The raw matrix file contained three matrices, including the “Multiplexing Capture,” a CMO read count matrix. We chose the cell-droplet barcodes that were listed in the Multiplexing Analysis result file above and that had more than one million CMO reads. Then we demultiplexed them with deMULTIplex2 (version 1.0.1) [33]. Because no droplets were classified as unlabelled cells, the partial stealth multiplet was not foreseen in theory, and we regarded this CMO-demultiplexed sample information as a reference.

Based on the deMULTIplex2 results, we built a custom VCF file containing each donor genotype. First, we merged all bam files (CMO301, CMO302, CMO303, CMO304, CMO306, CMO307, CMO308 and unassigned) that were pre-demultiplexed by 10x Genomics, sorted and indexed with SAMtools (version 1.17) [35]. Then, we subset the reads for each donor with subset-bam (version 1.1.0, 10x Genomics) with the cell barcode assigned by deMULTIplex2 results above. These subset bam files of each donor were subject to cellsnp-lite [36] to determine the genotype for the reference SNPs. As a reference, we used the human genome (hg38) SNP reference file with minor allele frequency $> 5 \times 10^{-4}$ from the Human variants from the 1000 genome project [36]. The collected individual genotypes of each donor were combined with BCFtools [35] and used as a reference VCF file for SNP-demultiplexing. SNP-demultiplexing was performed with demuxalot as described in the CITE-seq dataset section with the same posterior probability cut-off (0.5). In total, 106,556 SNPs were parsed from the custom reference file. To visualise the CMO count matrix, the CMO counts were standardised with the ScaleData function of Seurat, followed by PCA. UMAP was performed on all 7 PCs with the default settings of the Seurat RunUMAP function.

On the other hand, the gene expression matrix was stored as the “Gene Expression” matrix in the raw matrix file above. After selecting the barcodes of the cell-droplets, as is the case with the CMO matrix, we created a Seurat object from it. We normalised it with the NormalizeData function of Seurat and selected highly variable features with the FindVariableFeatures(selection.method = “mean.var.plot”) function. With these features, we standardised the dataset with the ScaleData function, and the resulting Seurat object was used as an input for the in silico doublet remover analysis described in a subsequent section.

#### Demultiplexing with Freemuxlet

We obtained a reference SNP list (in the VCF format) from the 1000 Genomes Project on GRCh38 [37] (ENA Accession: PRJEB30460) and filtered the SNPs located in gene bodies (coordinates from GENCODE release 32), and the ones with alternative allele frequency were >5%. In total, 4,144,050 SNPs were included in a primary SNP list for demultiplexing. This VCF file was further filtered with the read mapping file (BAM files) of corresponding datasets and sorted with the order of reads with popscle_helper_tools [38] before demultiplexing. Demuxafy [39] singularity image (version 3.0.0, available on Demuxfy GitHub repository (https://github.com/drneavin/Demultiplexing_Doublet_Detecting_Docs/blob/main/docs/source/Installation.rst)) was used to run Freemuxlet (an extension of demuxlet [17], version v0.1-beta) under Singularity (Apptainer) (version 1.3.6-1.el8). The CITE-seq dataset was remapped to the GRCh38 reference genome with cellranger count (10x Genomics) for Freemuxlet. The sample numbers were set as 9 and 7 for the CITE-seq and NSCLC datasets, respectively.

#### Downsampling

Downsampling was done with the SAMtools view -s command [35] with three different seeds. Each downsized bam file was sorted and indexed with the SAMtools sort and index commands, respectively, and demultiplexed with demuxalot with the same settings as the full read analysis above, including the thresholds for the posterior probability (0.5). These results were compared with the HTO-based (CITE-seq dataset) or CMO-based (NSCLC dataset) full-read demultiplexing reference results. We calculated the proportion of the four categories of multiplets by averaging the three replicates. The ratio of the Singlet to the monolabelled droplet (Supplementary Figs. 5D and 6D) was based on the “Singlet” definition in Supplementary Figs. 5B and 6B. Note that this ratio is different from *TSR*, which also considers the homogenous stealth multiplet that we cannot observe in the experimental settings in this study. To estimate the theoretical ratio of the four categories of multiplets in the downsized samples, we first calculated an average labelling efficiency from averaged observed counts of monolabelled and unlabelled droplets in each downsampling proportion. Then, the proportions of the four categories of multiplets were estimated under the fixed $\lambda =0.369$ and $\lambda =0.608$ for the CITE-seq and NSCLC datasets, respectively (See Supplementary Information Section 5 for details).

#### Multiplet detection by in silico doublet removers

We applied three in silico doublet removers, Scrublet, DblFinder, and DoubletFinder to the two-sample, CITE-seq, and NSCLC datasets. The expected doublet rates applied were 3.2%, 20.0%, and 16.0% for the two-sample, CITE-seq, and NSCLC datasets, respectively.

Scrublet (version 0.2.3 under Python 3.11.6) [8] through the R package scrubletR (version 0.1.0 under R version 4.1.2): The converted Seurat object of two-sample transcriptome data (see the “Clustering and visualisation” section for details) was input into scrubletR. For the CITE-seq, we used the Seurat object derived from the original feature count matrix. For the NSCLC datasets, the generated Seurat object was used as input.

The DblFinder package (version 1.16.0) [11] in R (version 4.1.2): We input the normalised SingleCellExperimet object of the two-sample dataset generated as described in the “Quality control of scRNA-seq” section above. For the CITE-seq and NSCLC datasets, the Seurat objects described above were converted with the as.SingleCellExperiment function in Seurat to SingleCellExperiment objects, which were used as inputs.

The DoubletFinder package (version 2.0.4) [9] in R (version 4.1.2): The Seurat object of the two-sample dataset was normalised, selected variable features, and standardised again with NormalizeData, FindVariableFeatures(selection.method = “vst”, nfeatures = 2000), and ScaleData functions in Seurat, respectively. PCA was also performed again because new variable features were selected. We used the doubletFinder function to discover doublets by setting the parameters PCs = 1:10, pN = 0.25 and pK = 0.18, reuse.pANN = FALSE, sct = FALSE. We chose pK = 0.18, which maximised the mean-variance normalised bimodality coefficient. Also, the nExp option was set based on the expected doublet rate, 3.2%. For the CITE-seq and NSCLC datasets, the generated Seurat object was used as input, and the doubletFinder function was used with the same parameters as the two-sample dataset, except pK, which was set to 0.010 and 0.005 for the CITE-seq and NSCLC datasets, respectively. These values were determined so that the pK maximised the AUC of the doublet/singlet classification when the SNP-based demultiplexing results were regarded as a reference. The nExp option was set to 20% and 16% based on the expected doublet rates for the CITE-seq and NSCLC datasets, respectively.

## Results

### Defining the four types of multiplets under the multiple sample conditions

To examine the composition of multiplets in mx-scRNA-seq, we need to consider the sample labelling status because inadequately labelled cells are potentially classified as unlabelled cells by the demultiplexing after sequencing. More specifically, we propose four categories of multiplets in mx-scRNA-seq from the viewpoint of sample barcoding: (I) the multiplet of all cells with the same sample barcode and detected as monolabelled (homogeneous stealth), (II) the multiplet that is a combination of a labelled cell(s) of single barcode and an unlabelled cell(s) and detected as monolabelled (partial stealth), (III) the multiplet which contains cells with different sample barcodes (multilabelled), and (IV) the multiplet consisting only of unlabelled cells (unlabelled) (Fig. 1A). While the last two categories, multilabelled multiplet and unlabelled multiplet, can be eliminated easily, the homogeneous and partial stealth multiplets are undetectable at the demultiplexing step. We also point out that the partial stealth multiplet was overlooked in previous reports [11, 14, 20, 21], probably because of the perceived low risk of unlabelled cells.

We used a Poisson distribution model [6, 9, 21, 40] and combinatorics (Fig. 1B and Supplementary Information Section 1 and 3 for details) to estimate the probability of these multiplets. Figure 1C-E show the theoretical fraction of multiplets in each category. For a fixed number of samples and a given Poisson distribution parameter $\lambda$, which represents the average number of cells per droplet, the proportion of unlabelled multiplets decreased as the labelling efficiency increased, while the proportion of multilabelled multiplets increased. Importantly, the proportion of partial stealth multiplets was greater than the multilabelled multiplets at low labelling efficiencies. Ideally, when the labelling efficiency was 1, the multiplets consisted of only the homogeneous stealth and multilabelled types (Fig. 1C). Interestingly, for a given labelling efficiency and $\lambda$, the homogeneous stealth multiplets were dramatically reduced as the number of samples increased, but the partial stealth type persisted because it was able to be formed with unlabelled cells from any other sample(s) (Fig. 1D, Supplementary Fig. 1). The value of $\lambda$ did not strongly alter the composition of the types of multiplets, though it affected the overall multiplet ratio (Fig. 1E). We defined the true singlet ratio (*TSR*) as the fraction of true singlets among monolabelled droplets. The *TSR* improved as the number of samples increased because of the effective removal of homogeneous stealth and multilabelled multiplets, but this effect diminished when the labelling efficiency was low because the partial type was the major stealth multiplet (Fig. 1F). Thus, the prediction here emphasises the importance of optimal labelling in mx-scRNA-seq.

### Partial stealth multiplets appeared in mx-scRNA-seq datasets with suboptimal labelling or sample demultiplexing

Next, we examined whether an actual mx-scRNA-seq dataset has any partial stealth multiplets, as we predicted. NIH3T3 (3T3) and mouse embryonic stem (ES) cells were labelled with distinct lipid-conjugated oligonucleotide barcodes before pooling so that transcriptome analysis alone easily identified multiplets across samples (providing “ground truth” data). In this dataset, we detected 1,645 cells, including 803, 784, and 58 cells in the 3T3, ES, and multiplet clusters, respectively (Fig. 2A). We tested three demultiplexing algorithms, the HTODemux function from the R package Seurat (Seurat::HTODemux) [30], GMM-demux [21], and the R package deMULTIplex [14], among others such as CITE-seq [12], BFF [41], demuxmix [42], and deMULTIplex2 [33]. In this dataset, the signal-noise ratio of ES cell barcode was not high enough to separate them into two distinct populations, i.e. positive and negative cells (Fig. 2B, Supplementary Fig. 2A). This indeed yielded many unlabelled droplets and affected the demultiplexing with barcode reads by GMM-demux (Fig. 2C) and by the other algorithms we tested (Supplementary Fig. 2B and C). Among the doublets defined by transcriptome analysis (Supplementary Fig. 2D), 63% of them were classified as monolabelled cells, indicating the presence of partial stealth multiplets (Fig. 2D). They also appeared irrespective of demultiplexing algorithms. (Supplementary Fig. 2E). In addition, our theoretical estimation of the partial stealth multiplets (2.81%) was consistent with the observed result (2.80%) (Supplementary Information Section 5). Interestingly, GMM-demux adopted a conservative threshold in the ES cell classification and failed to classify a substantial number of ES cells (Fig. 2C), leading to the misclassification of multiplets as 3T3 cells, which suggests that a conservative threshold in the suboptimal dataset can increase the risk of partial stealth multiplets.

We explored another dataset with very optimal labelling of three transcriptomically distinct samples (embryonic hindlimb bud mesenchyme (HL), ES, and 3T3 cells; Fig. 2E). In total, 2,697 cells (726, 498, and 1,473 cells in the HL, ES, and 3T3 cell clusters, respectively) were detected. This dataset had a clear bimodal distribution of barcode read per cell (Fig. 2F), and an appropriate demultiplexing algorithm, GMM-demux, was able to efficiently demultiplex the sample barcodes, resulting in only 19 unlabelled cells (Fig. 2G, Supplementary Fig. 3A-E). In this case, very few partial stealth multiplets were detected (2 in total; Fig. 2H, Supplementary Fig. 4A-D), which is also theoretically expected (Supplementary Information Section 5). Importantly, we detected more partial stealth multiplets with the other algorithms we tested (Supplementary Fig. 4B-D), indicating that the demultiplexing algorithm is another essential factor, and the risk of partial stealth multiplets can be reduced by choosing an adequate method for a given dataset. These results demonstrated the presence of partial stealth multiplets in actual mx-scRNA-seq. As theoretically predicted, the ratio of partial stealth multiplets largely depends on the sample labelling efficiency, which is determined not only by the sample labelling step but also by the choice of demultiplexing algorithm.

**Fig. 3:**
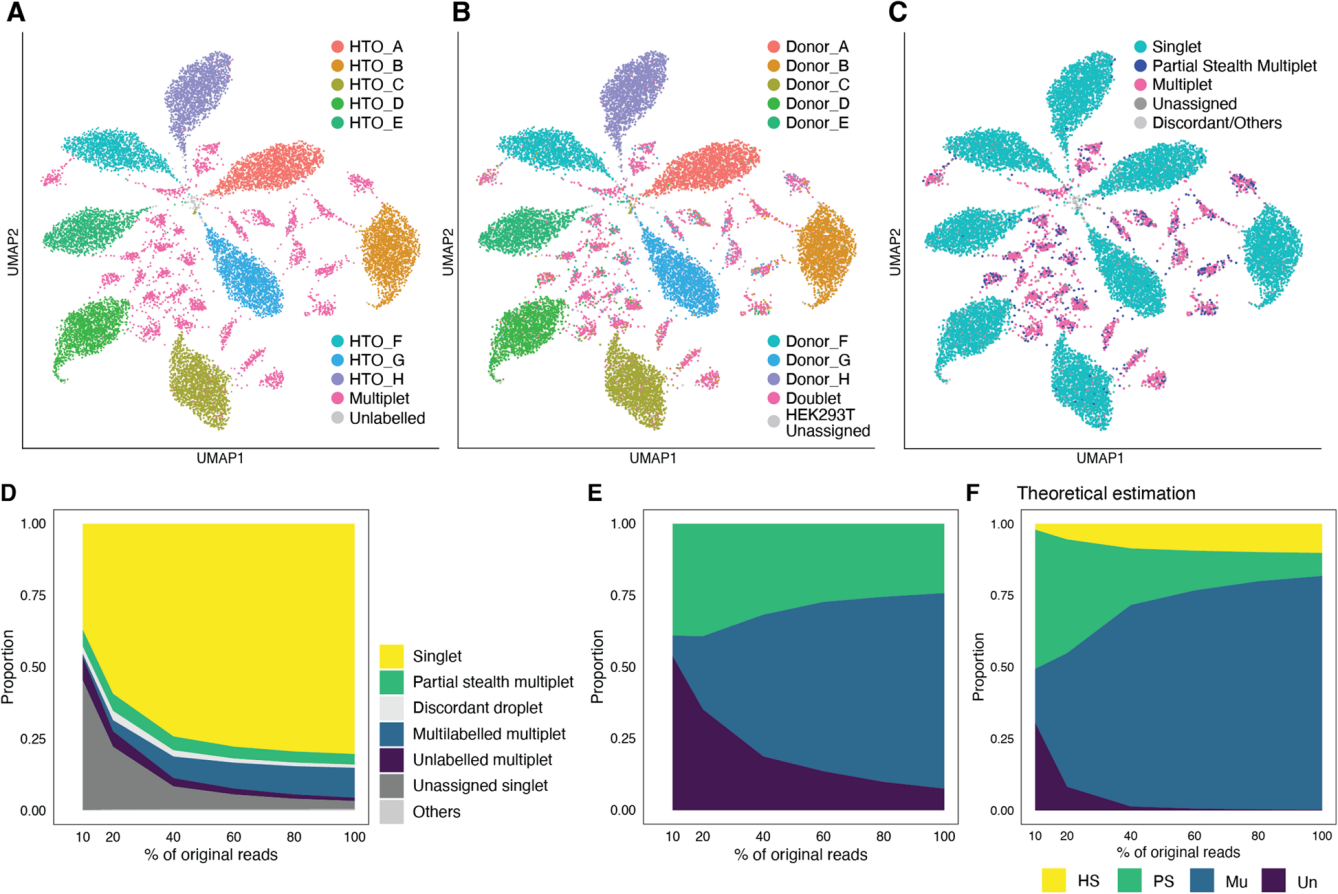
Partial stealth multiplets appeared in the PBMC CITE-seq dataset when demultiplexed with SNPs. **A** A UMAP plot of the barcode read matrix of the CITE-seq dataset [20] multiplexing PBMC samples from 8 donors and HEK293T cells, which were mostly removed before this analysis. **B** The UMAP plot, same embeddings as (**A**), is colour-coded by the SNP-demultiplexing results with demuxalot [34]. **C** The UMAP plot, same embeddings as (**A**), compares SNP-demultiplexing by demuxalot and the “ground truth” HTO-demultiplexing results. A schematic diagram of this classification is shown in Supplementary Fig. 5B. **D** Proportion of each type of droplet in a downsampling analysis. An average of three replicates of downsampled reads was plotted. The droplet classes were defined as shown in Supplementary Fig. 5B. **E** The proportions of partial stealth, multilabelled, and unlabelled multiplets in the downsampling experiment, based on Fig. 3D. An average of three replicates was shown. **F** Theoretical estimation of the proportion of four types of multiplets in the downsampling experiment. We estimated a labelling efficiency for each downsampling proportion with the fixed $\lambda =0.369$, inferred from the observed multilabelled droplet count by deMULTIplex2. HS, Homogeneous stealth, PS, Partial Stealth, Mu, Multilabelled, and Un, Unlabelled

### Partial stealth multiplets appeared in SNP-based demultiplexing

The previous experiments illustrated the presence of partial stealth multiplets in the relatively simple mx-scRNA-seq datasets that can easily delineate them. Still, it is unlikely that a similar setting is applicable in other experiments. To further examine whether the partial stealth multiplet exists in the dataset of standard settings—multiplexing more samples and similar cell types, we sought to analyse two more datasets that could be demultiplexed by either oligonucleotide barcodes or SNPs so that we have two different types of information from which to assess partial stealth multiplets. Depending on which of these gives more reliable results, we can then use that information as the baseline to assess the reliability of the other approach.

The first dataset was the CITE-seq dataset [20], containing peripheral blood mononuclear cells (PBMCs) from 8 donors multiplexed after labelling with Hashtag oligonucleotides (HTOs). The demultiplexing result with deMULTIplex2 [33] based on HTO reads is shown in Fig. 3A. The eight large clusters represent the droplets containing a cell from each donor, and numerous small clusters represent multiplets containing more than one barcode species. The number of cells were 1,918, 2,001, 1,876, 1,726, 1,493, 1,545, 1,833, 1,874, 2,578, and 72 for the HTO_A, HTO_B, HTO_C, HTO_D, HTO_E, HTO_F, HTO_G, HTO_H, Multiplet, and Unlabelled, respectively. Because the samples were very well labelled, only a few unlabelled droplets were detected, which suggests the risk of partial stealth multiplet is <0.2% based on our proposed model (Supplementary Information Section 5). The same dataset was demultiplexed with demuxalot [34] based on the donor genotype information, and 96% of droplets were successfully assigned. However, multiplets comprised only 10% of the droplets. This is fewer than the ratio of multiplet detected with HTO (15%), let alone the expected ratio (20%) (Fig. 3B and C, Supplementary Fig. 5A). Thus, we regarded HTO-demultiplexed results as the “ground truth” data and assess the concordance of the two demultiplexing results as follows: “Singlet” is a droplet with a singlet call of an identical sample by demuxalot and deMULTIplex2. “Partial stealth multiplet”, a singlet call by demuxalot but a multiplet call by deMULTIplex2. “Multiplet”, a multiplet call by demuxalot and deMULTIplex2. “Unassigned”, an unassigned droplet by demuxalot. The others were included in “Discordant/Others”. According to this classification, the number of Singlets, Partial stealth multiplet, Multiplet, Unassigned, and Discordant/Others were 13,588, 624, 1,759, 680, and 265, respectively. In fact, these many partial stealth multiplets in the SNP-based demultiplexing result appeared as singlets in HTO-multiplet clusters (Fig. 3C, Supplementary Fig. 5A).

Although SNPs are intrinsic sample markers, the sequencing depth may affect their detection. Hence, the demultiplexing performance and the ratio of the four types of multiplets may also be affected. Indeed, when we downsampled the sequence reads, the number of unassigned cells increased (Fig. 3D, Supplementary Fig. 5C), and the ratio of singlets to monolabelled droplets dropped (Supplementary Fig. 5D). This mimicked the low labelling efficiency in the oligonucleotide-barcode-based demultiplexing and highlighted the significance of sequencing depth in SNP-based mx-scRNA-seq. Interestingly, however, the partial stealth multiplets remained more than expected even in the whole read analysis when we theoretically estimate the proportion of the four categories of multiplets (Fig. 3E and F. See Methods and Supplementary Information Section 5 for details on estimation). In other words, the number of multilabelled multiplets did not rise as predicted by the Poisson distribution model (Fig. 3F).

**Fig. 4:**
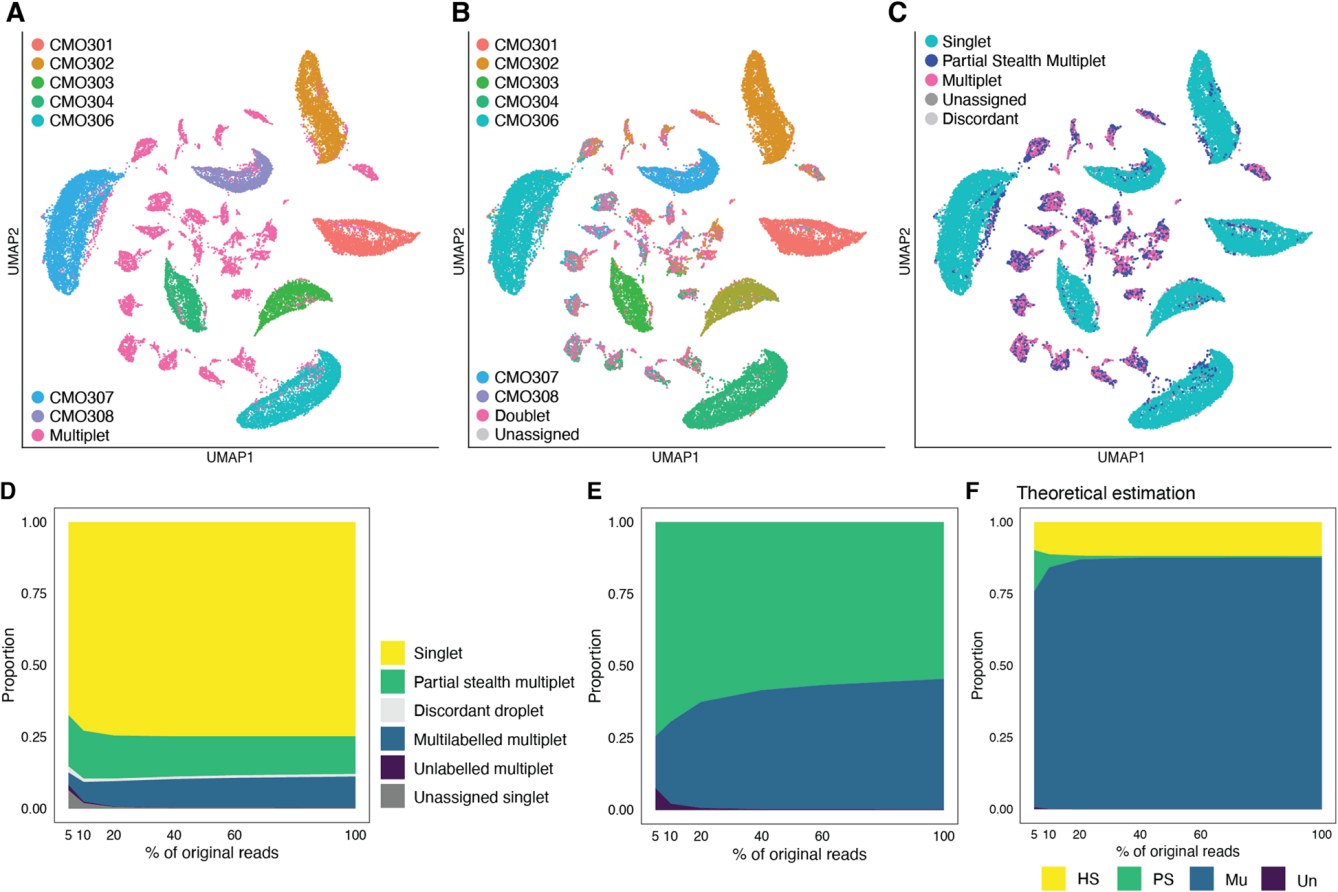
Partial stealth multiplets appeared in the NSCLC Total-seq dataset when demultiplexed with SNPs. **A** (**A**-**C**) Similar to Fig. 3A-C in the case of the 40k NSCLC Total-seq dataset (10x Genomics). **A** CMO-based demultiplexing with deMULTIplex2 is shown. **B** SNP-based demultiplexing with demuxalot is shown. **C** The SNP-based demultiplexing performance in (**B**) is compared with the reference in (**A**). (**D**-**F**) Similar to Fig. 3D-F. **D** The proportion of each droplet class is shown. For (**C**) and (**D**), a schematic diagram of the classification is shown in Supplementary Fig. 6B. **E** Based on (**D**), the proportions of partial stealth, multilabelled, and unlabelled multiplets are shown. **F** Theoretical estimation of the proportion of the four categories of multiplets. $\lambda =0.608$ was used for this estimation

These analyses were applied to another dataset, the 40k non-small cell lung cancer (NSCLC) Total-seq dataset (10x Genomics), which multiplexed the samples from 7 donors and was able to be demultiplexed with either the Cell Multiplexing Oligos (CMOs) or donor genotype. As seen previously in the CITE-seq detaset, the CMO-based demultiplexing gave better performance (Fig. 4A and B). The numbers of cells assigned by deMULTIplex2 were 3,208, 3,844, 2,243, 2,108, 4,840, 6,220, 2,342, and 7,864 for the CMO301, CMO302, CMO303, CMO304, CMO306, CMO307, CMO308, and Multiplet, respectively. Interestingly, however, although both of the demultiplexing methods successfully classified most cells, deMULTIplex2 identified more multiplets than demuxalot (Fig. 4B and C, Supplementary Fig. 6A). Consequently, the number of Singlets, Partial stealth multiplet, Multiplet, Unassigned, and Discordant were 24,433, 4,285, 3,561, 88, and 302, respectively (Fig. 4C, Supplementary Fig. 6A), indicating approximately 15% of monolabelled cells are partial stealth multiplets in the SNP-based demultiplexing results. The downsampling analysis revealed persistent partial stealth multiplets and a decrease in the ratio of singlets among monolabelled droplets (Fig. 4D and E, Supplementary Fig. 6C and D), consistent with the results in the CITE-seq dataset. Again, the number of multilabelled multiplets was not as many as theoretically predicted (Fig. 4C) (Methods and Supplementary Information Section 5 for details on estimation). We also observed this under-detection of doublets with another SNP-based demultiplexing algorithm, Freemuxlet [17]. Although it assigned practically all droplets (> 99.9%), it detected only 30–40% of doublets determined by external labels and classified the rest as singlets (i.e. partial stealth multiplets) (Supplementary Fig. 7).

We also tested in silico doublet removers to see whether they can eliminate partial stealth multiplets. In the two-sample dataset, they successfully removed 68%−85% of the partial stealth multiplet, depending on the algorithms (Supplementary Fig. 8A). Given that the two samples in this dataset are very distinct cell types, this will be favourable for these algorithms, suggesting that their success rates shown here may not be applicable to the typical datasets in which cell type composition of the multiplexed samples are similar to each other even though each sample consists of heterogenous cell populations. In this case, in silico doublet removers are expected to work well for homogeneous stealth multiplets consisting of cells from the same sample. However, once they are multiplexed, it is challenging to detect multiplets consisting of similar cells across samples, regardless of heterogeneity within each sample. Indeed, when in silico doublet removers were applied to the CITE-seq (Supplementary Fig. 8B and C) and NSCLC datasets (Supplementary Fig. 8D and E), the algorithms were significantly less successful: only 32% and 39% of partial stealth multiplets, at best, were removed, respectively. i.e. their majority still remain. In the NSCLC dataset, the partial stealth multiplet still occupied >10% of the singlets classified by SNP-based demultiplexing even after the application of doublet removers (Supplementary Fig. 8D). Therefore, we concluded that these doublet removers will not always be a remedy for the partial stealth multiplet in a common setting in which samples with very similar cell type compositions are multiplexed.

## Discussion

This article provides the first theoretical analysis, as far as we are aware, of the four types of multiplets that occur in mx-scRNA-seq: Homogeneous stealth, partial stealth, multilabelled, and unlabelled multiplets. Among them, the multilabelled and unlabelled multiplets can be excluded as multilabelled and unlabelled cell-droplets. In contrast, the homogeneous stealth and partial stealth multiplets cannot be unambiguously detected because they appear as monolabelled cell-droplets. We examined their probability based on the Poisson distribution model. As a result, both types of multiples depend on the labelling efficiency and $\lambda$, the mean occurrence of cell-droplet formation per droplet.

The first key parameter, the predicted labelling efficiency, is determined mainly by two factors: One is the sample labelling process, and the other is the choice of an in silico demultiplexing algorithm. Certainly, optimising the labelling process to maximise the signal-to-noise ratio is essential. On the other hand, whether these labelled cells are classified as label-positive depends on how the algorithm differentiates the positive and negative populations. In other words, the demultiplexing algorithm *per se* defines the apparent labelling efficiency by cutting off the continuous barcode read counts. Therefore, choosing an appropriate demultiplexing method is another critical step in mx-scRNA-seq.

It is important to demultiplex the cells with an algorithm based on an appropriate sample-barcode read distribution model. In our case, GMM-demux was optimal for the well-labelled dataset, which is probably because the dataset contains only three samples with sufficient read depth to capture the bimodal log-standard distributions. By contrast, deMULTIplex2 [33], which is one of the latest algorithms and applied to SNP-based demultiplexing datasets in this study, was not applicable because our datasets violate their assumption in terms of the cell size variation among samples (Qin Zhu, personal communication), which emphasises the importance of exploring an appropriate method utilising a relevant assumption for a given dataset.

The other key parameter, $\lambda$, which is correlated with cell loading rates onto a microfluidic device, determines the overall multiplets ratio. Therefore, loading fewer cells can reduce the risk of overall multiplets. However, the recent trend in mx-scRNA-seq is increasing the number of loading cells by expecting effective multiplet removal [14, 20]. Thus, the model and prediction here will be a caveat against the suboptimally labelled mx-scRNA-seq, especially when an excessive number of cells are “super-loaded.” In fact, unlike homogeneous stealth multiplets and multilabelled multiplets, increasing the number of sample barcodes cannot reduce partial stealth multiplets. Moreover, in silico doublet removers were also unable to effectively remove them. Therefore, high-quality control of the mx-scRNA-seq, that is, appropriate sample labelling with a sufficient signal-to-noise ratio in vitro and adequate sample demultiplexing in silico, is critical for circumventing the nuisance of hidden multiplets.

We extended our analysis to SNP-based demultiplexing and still found partial stealth multiplets. Our results exemplified that SNP-based demultiplexing often outperformed in terms of the number of droplets assigned, but is less sensitive in detecting doublets than external-label-based methods. Therefore, it did not follow our Poisson distribution model-based prediction. This discrepancy can be explained by the SNP-demultiplexing method: Multiplets are not predicted as a droplet with high possibilities of multiple samples, but instead directly assigned by estimating the posterior probability of each doublet combination [34], which may under-detect the multilabelled multiplet and lead to a greater risk of stealth multiplets.

In fact, the original report of the CITE-seq dataset [20] compared the HTO-demultiplexing results with SNP-based demultiplexing by demuxlet [17] and indicated a certain proportion of HTO-doublets were assigned to singlets by demuxlet. Another report comparing CMO-based demultiplexing with an SNP-based one by Souporcell [43] also indicated that a substantial proportion of CMO-multiplets were classified as singlets [44]. These SNP-based algorithms, especially demuxalot and Souporcell, were the first choices in an extensive benchmark study [39]. Thus, the possibility of multiplet under-detection should be noted in SNP-based demultiplexing. In order to mitigate the risk of stealth multiplets, we propose that it is preferable to detect as many multilabelled multiplets as expected by the experimental settings rather than only focusing on reducing the number of unassigned cells.

We modelled the probabilities of multiplets in scRNA-seq based on the Poisson distribution and combinatorics by assuming randomness and low frequency in the cell-droplet formation process. However, there may be some minor deviations from this assumption; The multiplet cells that contacted each other due to incomplete dissociation violate the randomness assumption and distort the multiplet ratio. Also, massive loading of cells violates the low-frequency assumption. Indeed, it is reported that the negative binomial distribution better explained the actual cell-droplet formation in such a case [45]. Another source of errors is the beads for capturing transcripts. Bead integration is much more frequent than that of cells, but it does not always happen, which leads to an error in estimating the multiplet ratio because we can observe cells only when a bead exists in the same droplet. According to a report [46], 16.1%, 80.0%, and 3.9% of droplets had 0, 1, and $\le$2 beads, respectively, with the Chromium Controller (10x Genomics).

A drawback of the application of the Poisson distribution to actual mx-scRNA-seq experiments is that estimating the key parameter $\lambda$ in routine experimental settings is challenging. Although $\lambda$ was estimated from the specification of a microfluidic device in this study, it may be inaccurate due to technical variations of devices and experiments. Nonetheless, the Poisson distribution model can concisely describe the process of cell-droplet formation and is useful for estimating the probability of each type of multiplets within a range of cell loading rates in most mx-scRNA-seq experiments.

## Conclusions

We have theoretically demonstrated four types of multiplets in mx-scRNA-seq: Homogeneous stealth, partial stealth, multilabelled, and unlabelled multiplets. Among them, the partial stealth multiplet, which consists of a monolabelled cell(s) with an unlabelled cell(s), has not previously been scrutinised. We have developed a theoretical approach to quantify this category, experimentally illustrated their existence in oligonucleotide-barcoded and SNP-based mx-scRNA-seq datasets, and found two types of issues that may lead to partial stealth multiplets being significant. Firstly, insufficient labelling in vitro complicates the subsequent demultiplexing and increases the number of unlabelled or unassigned droplets. This can be due to either suboptimal external labelling or insufficient read depth. Secondly, we reveal that the choice of the demultiplexing algorithm remarkably affects the estimation of partial stealth multiplets. Each algorithm has made its own assumptions, and choosing the one that suits the dataset of interest is critical. When using SNP-based algorithms, we found that they tended to under-detect multiplets and assign them to one of the samples instead, leading to an increase in the number of partial stealth multiplets. Therefore, we propose that the number of multiplets detected as well as the number of droplets assigned be used as criteria to evaluate the demultiplexing performance in mx-scRNA-seq.

## Supplementary Information

Supplementary Material 1.

## Data Availability

The mx-scRNA-seq datasets generated and analysed in this study are available in BioStudies (Accession No. E-MTAB-13181). The sample-barcode count matrices of datasets above and the R scripts for estimating the multiplet probabilities used in this study are available in GitHub (https://github.com/fnakaki/stealth-multiplet-notebook). The CITE-seq dataset (stoeckius et al. 2018) original read files are available at Gene Expression Omnibus (Accession No. GSE108313), and the original feature count matrix and HTO count matrix are available in a vignette for the R package Seurat, “Demultiplexing with hashtag oligos (HTOs)” (https://satijalab.org/seurat/articles/hashing_vignette). The 40k Mixture of NSCLC DTCs from 7 donors, 3’ HT v3.1 dataset (10x Genomics) is available at the 10x Genomics dataset repository (https://www.10xgenomics.com/datasets/40-k-mixture-of-nsclc-dt-cs-from-7-donors-3-ht-v-3-1-3-1-high-6-1-0).

## Abbreviations

scRNA-seq: Single-cell RNA sequencing (in this article, we use the term “scRNA-seq” specifically for microfluidic-based single-cell RNA sequencing)
mx-scRNA-seq: Sample-multiplexing scRNA-seq
TSR: True singlet ratio
HTO: Hashtag oligonucleotide
CMO: Cholesterol-conjugated oligonucleotide (in MULTI-seq) or Cell Multiplexing Oligos (in 3’ CellPlex)
PBMC: Peripheral blood mononuclear cell
NSCLC: Non-small cell lung cancer
UMAP: Uniform manifold approximation and projection
