# Supplementary material for: Probability of stealth multiplets in sample-multiplexing for droplet-based single-cell analysis
Source: BMC Genomics. 2025 Jul 23;26:686. doi: 10.1186/s12864-025-11835-z (PMC12285059; doi:10.1186/s12864-025-11835-z)
Supplement: Supplementary file 1 — Supplementary Material 1. [file 12864_2025_11835_MOESM1_ESM.pdf]

Supplementary Material

for "Probability of stealth multiplets in sample-multiplexing for droplet-based single-cell analysis"

Supplementary Figures

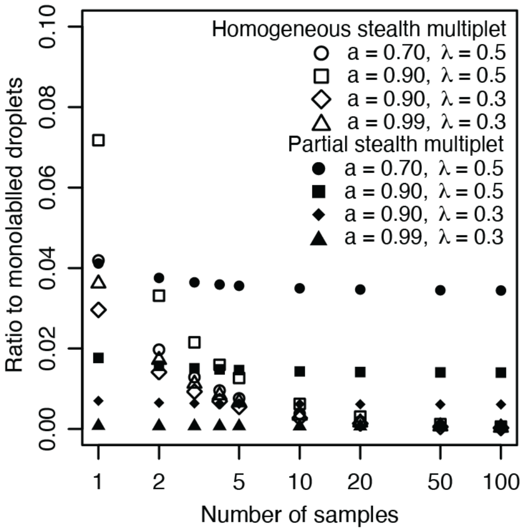

Supplementary Figure 1: Theoretical probability of stealth multiplets in a sample-multiplexed experiment The ratio of homogeneous stealth and partial stealth multiplets in monolabelled cell-droplets. Note that the partial stealth multiplet persists even when 100 samples are multiplexed.

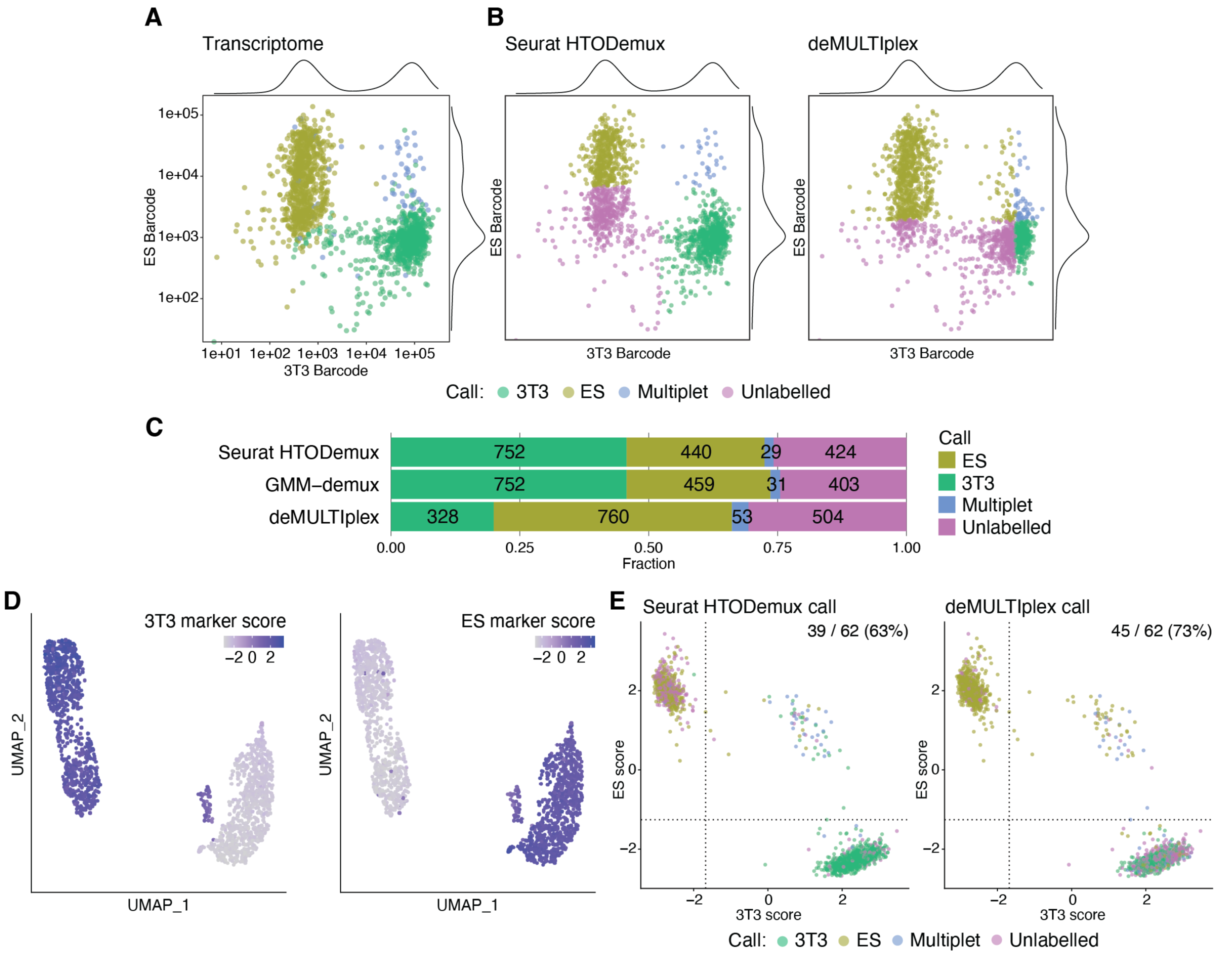

Supplementary Figure 2: The comparison between cell-type classification by transcriptome and by barcode read demultiplexing in a suboptimally labelled dataset (A) A log-transformed scatter plot of the barcode reads, colour-coded by clusters based on the transcriptome. Each barcode density plot (similar to the histogram in **Figure 2B** is also shown along each axis. (B) Similar to **Figure 2C**, the classification results of Seurat::HTODemux or deMULTIplex are projected on the same scatter plots as (A). (C) Summary of the classification results by the three different demultiplexing algorithms. The cell counts of each group are also shown. (D) To explore whether the partial stealth multiplet occurs, we defined the ES and 3T3 transcriptome scores based on their marker genes. The levels of the 3T3 (left) and ES (right) cell marker scores are shown in a UMAP plot of the transcriptome space. As expected, each population had a high score for its own lineage marker set but not the other, and the multiplet cluster highly expressed both markers. (E) Scatter plots of the 3T3 and ES scores same as **Figure 2D** but demultiplexed with Seurat::HTODemux or deMULTIplex. The cells in the right upper quadrant were regarded as the multiplets. The number of cells classified as monolabelled droplets among the multiplets (i.e. partial stealth multiplets), the total number of multiplets, and their percentage are shown at the top right.

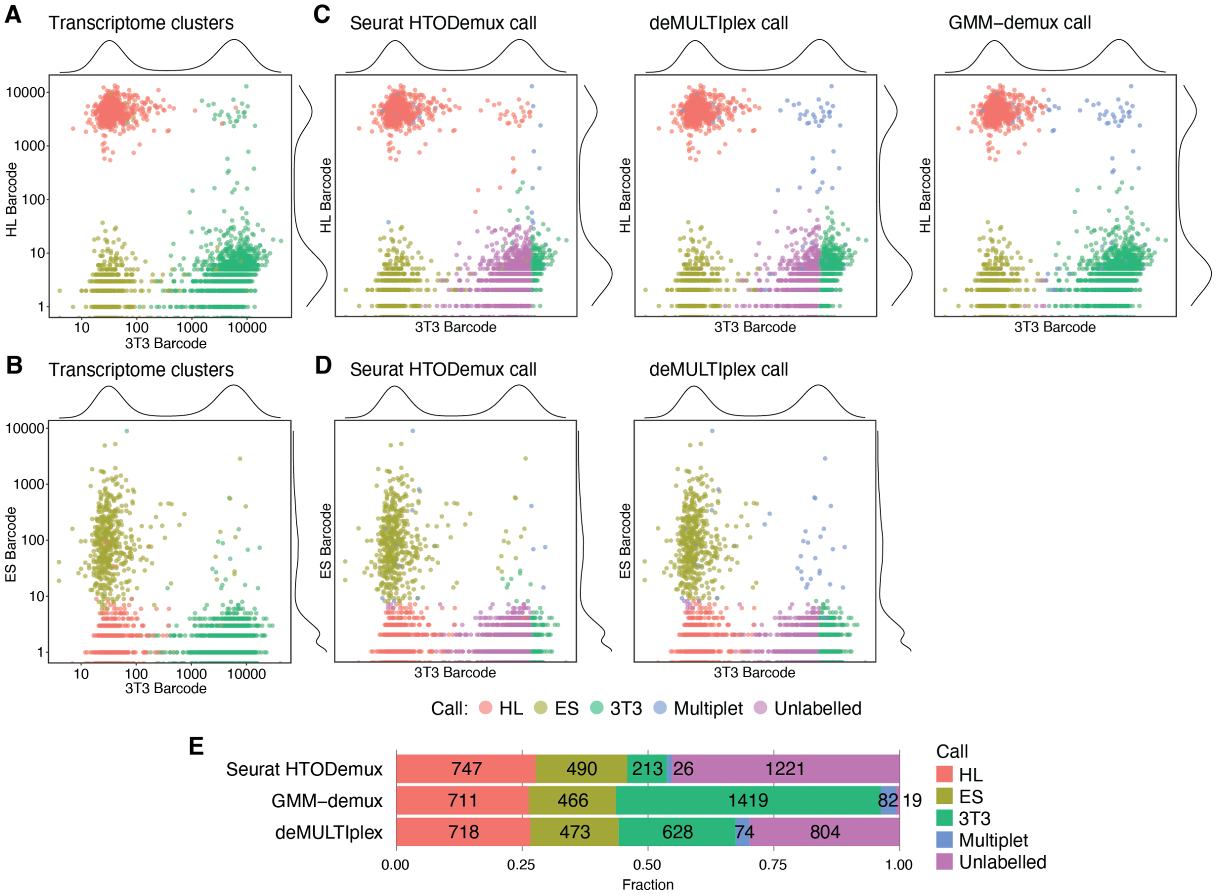

Supplementary Figure 3: The comparison between cell type classification by transcriptome and barcode read demultiplexing in an optimally labelled dataset (A, B) A log-transformed scatter plot of the 3T3 and HL barcode reads (A) and the 3T3 and ES barcode reads (B), colour-coded by the cluster based on the transcriptome. Each barcode density plot (similar to the histogram in **Figure 2F**) is also shown along each axis. (C, D) The classification results of each method are projected on the same scatter plot as (A) and (B). (E) Summary of the classification results by the three different demultiplexing algorithms. The cell counts of each group are also shown.

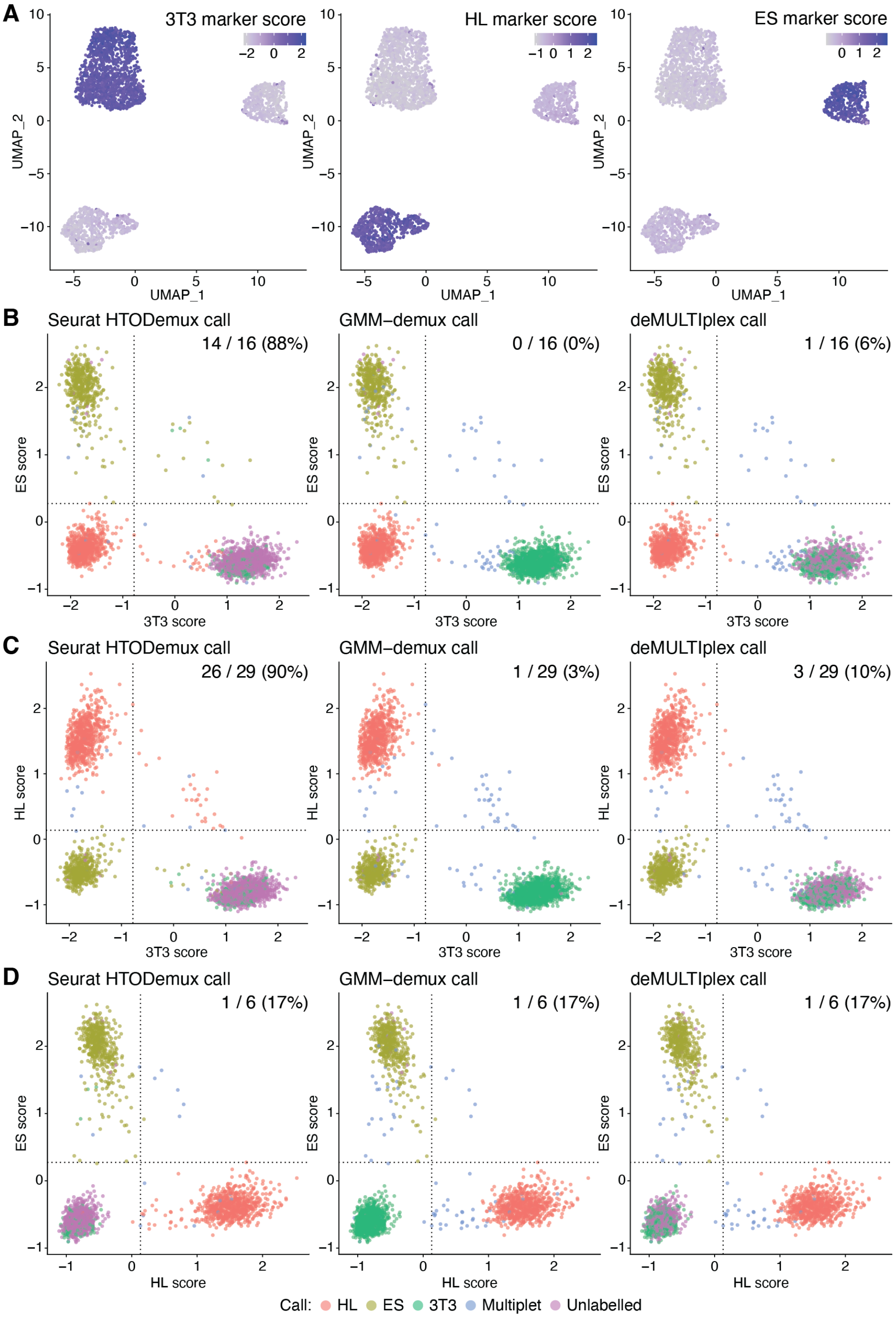

Supplementary Figure 4: Few partial stealth multiplets appear in an optimally labelled dataset with an appropriate demultiplexing method (A) The levels of the 3T3, HL, and ES cell lineage marker scores are shown on the UMAP plot in **Figure 2E**. Notably, several cells showed high scores in other lineage clusters, suggesting they were multiplets with cells of different lineages. (B-D) Scatter plots of the 3T3 and ES scores (B), the 3T3 and HL scores (C), and the HL and ES scores (D). The cells in the right upper quadrant were regarded as the multiplets. The number of cells classified as monolabelled droplets among the multiplets (i.e. partial stealth multiplets), the total number of multiplets, and their percentage are shown at the top right. The plot of the 3T3 score against ES score demultiplexed with GMM-demux (B, middle) is identical to **Figure 2H** but shown here as well for clarity.

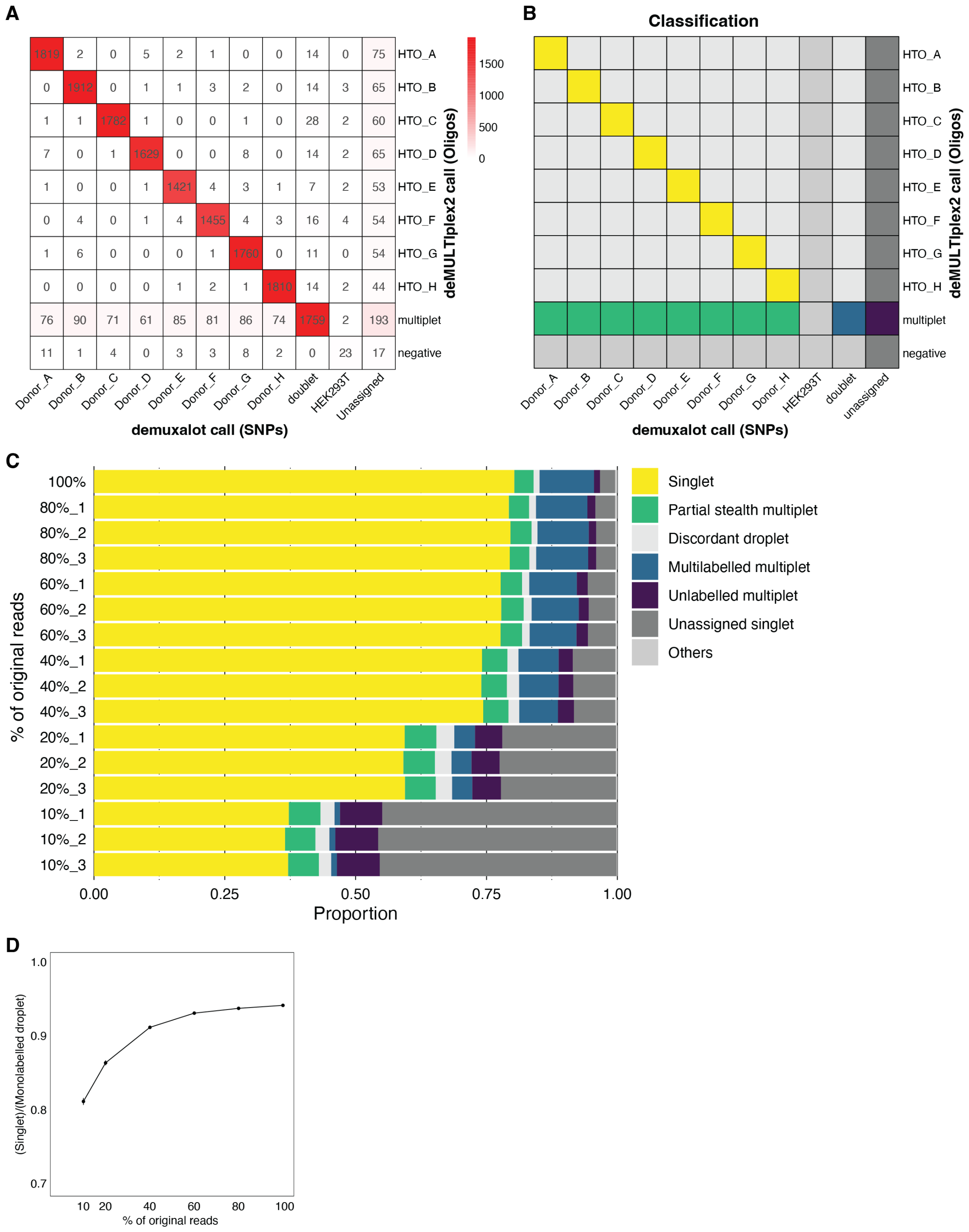

Supplementary Figure 5: Partial stealth multiplets by SNP-demultiplexing in the CITE-seq dataset (A) A contingency table to exhibit the concordance of the demultiplexing results by deMULTIplex2 (based on HTO reads) and by demuxalot (based on SNPs). The number of droplets in each combination is shown. (B) The definition of the classification of droplets shown in **Figures 3C and 3D**. The colour code of each combination of deMULTIplex2 and demuxalot calls is common to the figure legend of (C). Note that the Unlabelled singlet and multiplet were not distinguished in **Figure 3C**. (C) Proportions of droplets in each downsampled replicate. The average of three replicates is shown in **Figures 3D and 3E**. (D) The ratio of Singlets among the monolabelled droplets, including Singlets, Partial stealth multiplets, and Discordant droplets in the downsampled analysis. Mean±SD of three replicates are shown. Note that this index is similar to *TSR*, but we cannot observe the homogenous stealth multiplet in the actual data and, thus, did not consider them here.

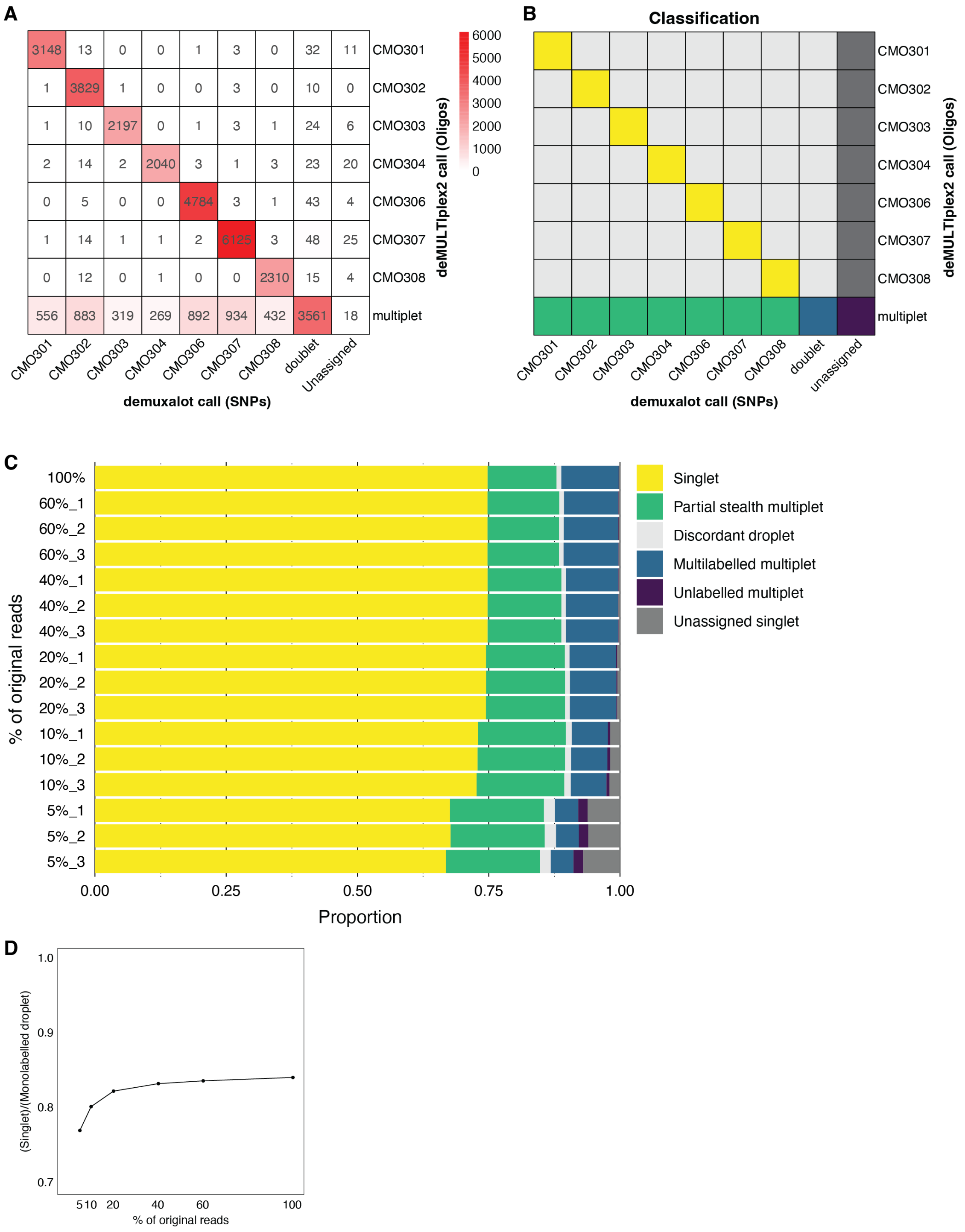

Supplementary Figure 6: Partial stealth multiplets by SNP-demultiplexing in the 40k NSCLC Total-seq detaset (A) A contingency table to exhibit the concordance of the demultiplexing results by deMULTIplex2 (based on CMO reads) and by demuxalot (based on SNPs). The number of droplets in each combination is shown. (B) The definition of the classification of droplets shown in **Figures 4C and 4D**. The colour code of each combination of deMULTIplex2 and demuxalot calls is common to the figure legend of (C). Note that the Unlabelled singlet and multiplet were not distinguished in **Figure 4C**. (C) Proportions of droplets in each downsampled replicate. The average of three replicates is shown in **Figures 4C and 4D**. (D) The ratio of Singlets among the monolabelled droplets, including Singlets, Partial stealth multiplets, and Discordant droplets in the downsampled analysis. Mean±SD are shown.

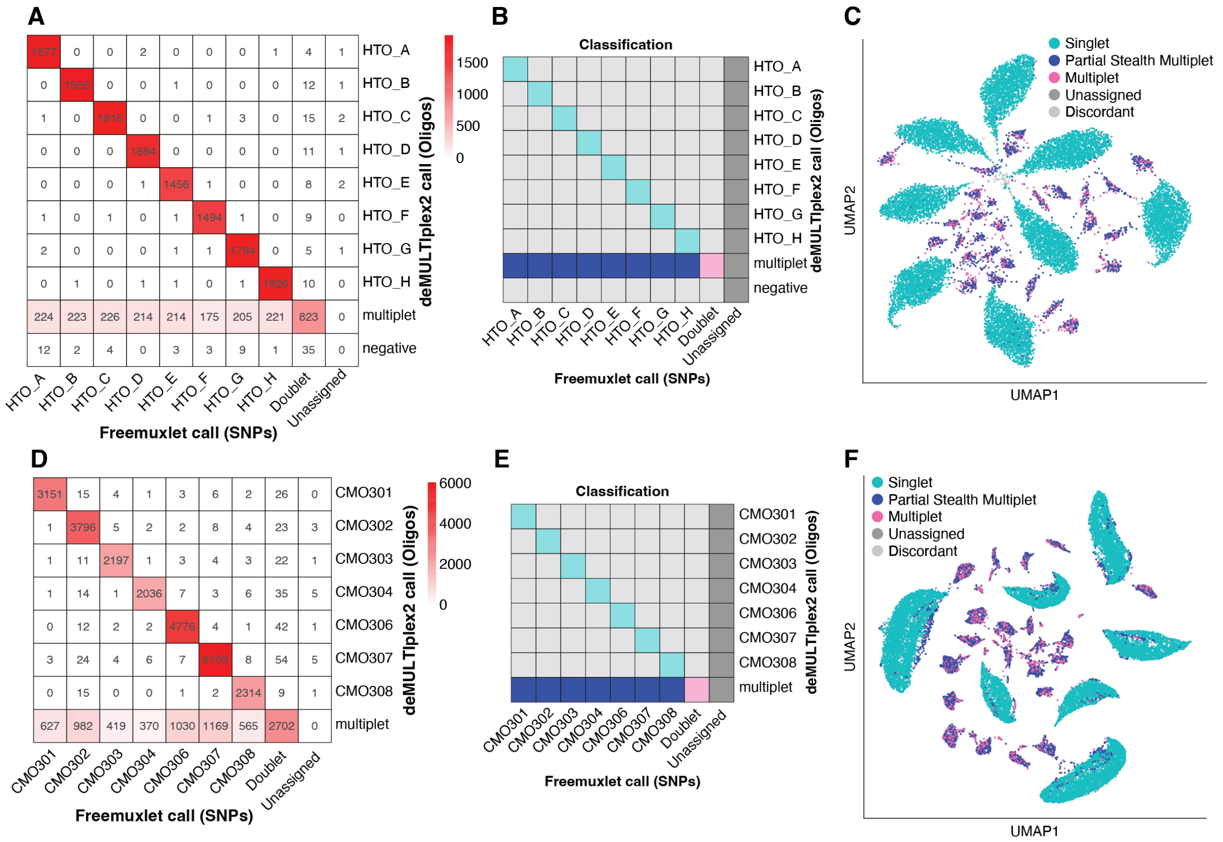

Supplementary Figure 7: Partial stealth multiplets appeared when using a different SNP-based demultiplexing algorithm (A) A contingency table to exhibit the concordance of the demultiplexing results of the CITE-seq dataset by deMULTIplex2 (based on HTO reads) and by Freemuxlet (based on SNPs). The number of droplets in each combination is shown. (B) The definition of the classification of droplets shown in (C). (C) The same UMAP embeddings as **Figure 3C**, but colour-coded based on the combination of Freemuxlet and HTO-demultiplexing results as shown in (B). Partial stealth multiplets are shown in blue. The numbers of Singlet, Partial stealth multiplet, Multiplet, Unassigned, and Discordant droplets were 13,902, 1,702, 823, 8, and 166, respectively. (D-F) The same analysis was performed on the NSCLC dataset. The numbers of Singlet, Partial stealth multiplet, Multiplet, Unassigned, and Discordant droplets were 24,379, 5,162, 2,702, 16, and 410, respectively.

A

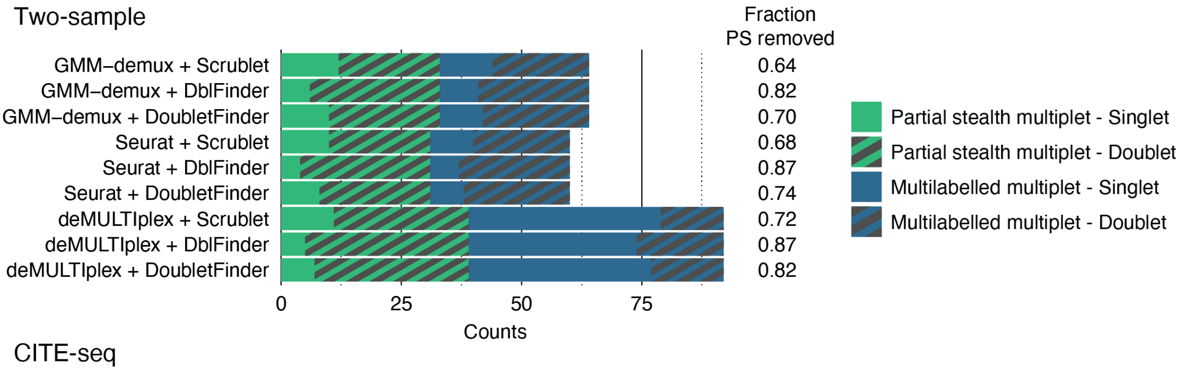

B CITE-seq

Algorithm	Singlet		Partial stealth		Multiplet		Unassigned		Discordant		Others	
	Singlet	Doublet	Singlet	Doublet	Singlet	Doublet	Singlet	Doublet	Singlet	Doublet	Singlet	Doublet
Scrublet	13200	388	507	117	1027	732	623	57	180	13	71	1
DblFinder	13044	544	472	152	463	1296	617	63	172	21	72	0
DoubletFinder	11589	1999	411	213	841	918	509	171	152	41	31	41

C

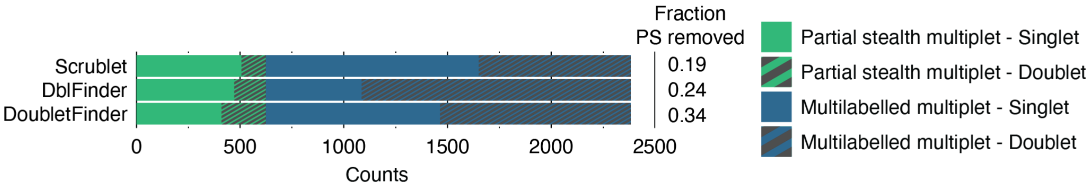

D NSCLC

Algorithm	Singlet		Partial stealth		Multiplet		Unassigned		Discordant	
	Singlet	Doublet	Singlet	Doublet	Singlet	Doublet	Singlet	Doublet	Singlet	Doublet
Scrublet	24395	38	4246	39	3275	286	82	6	285	17
DblFinder	22353	2080	2630	1655	1333	2228	88	0	183	119
DoubletFinder	22712	1721	3032	1253	1420	2141	84	4	194	108

E

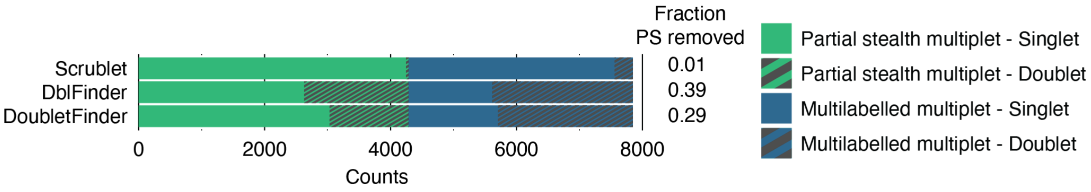

Supplementary Figure 8: *in silico* doublet removers did not detect partial stealth multiplets efficiently in the datasets that multiplexed similar cell types (A) The results of *in silico* doublet removers for the two-sample dataset are shown by combinations of algorithms. Partial stealth multiplets were identified by the transcriptome score analysis, and multilabelled multiplets were classified by each demultiplexing algorithm. Doublets identified by doublet removers are shown as bars with stripes. The fraction of partial stealth multiplest removed by each *in silico* doublet remover is shown on the right (Fraction PS removed). The average fractions of the removed partial stealth multiplet were 0.68, 0.85, and 0.75 for Scrublet, DblFinder, and DoubletFinder, respectively. (B) A summary of the results by *in silico* doublet removers in the CITE-seq dataset. The classification of droplets corresponds to **Figure 3C**. (C) A bar plot of the partial stealth multiplet and the multilabelled multiplet, extracted from the table (B). Doublets identified by doublet removers are shown as the bars with stripes. The fraction of partial stealth multiplet removed by each *in silico* doublet remover is shown on the right (Fraction PS removed). (D) The results of doublet detection with *in silico* doublet removers in the NSCLC dataset, grouped by the classification in **Figure 4C**. (E) A similar bar plot to (C) in the NSCLC dataset.

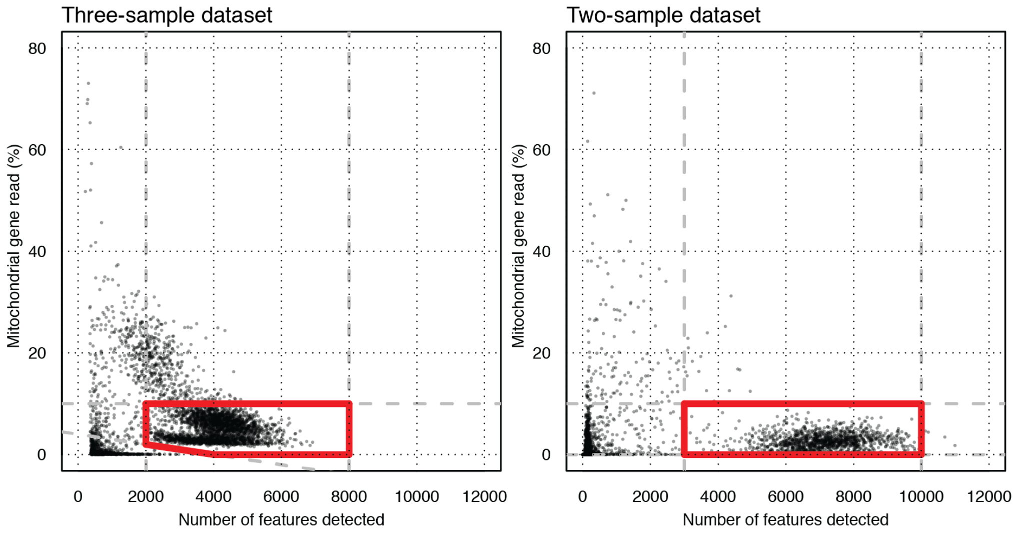

Supplementary Figure 9: The QC filters for scRNA-seq Scatter plots showed the number of features detected and the ratio of mitochondrial gene reads (%). The cells plotted inside the red polygons were filtered for further analysis.

Supplementary Information

1 Modelling a multiplet probability by the Poisson distribution

The probability of a droplet containing at least one cell (a cell-droplet) that contains a given number, k, of cells can be assumed to follow the Poisson distribution [1–4],

$$P(k) = \frac{\lambda^k e^{-\lambda}}{k!} \qquad (0 < \lambda \lesssim 1)$$

where λ is the average count of the event, representing the average cell numbers per droplet, which must be $0 < \lambda \lesssim 1$ in practice because cells should be loaded with low concentration to generate multiplets as few as possible, which also means λ is closely correlated with cell loading rates. Indeed, the scRNA-seq cell-droplet formation process, in which a cell enters into a droplet, meets the assumptions of the Poisson distribution that are A) random and B) low frequency. The situation that violates these assumptions is possibly a limitation of this model, and we discuss some deviations from this assumption in **Discussion**.

With this model, the probabilities of empty droplets, singlets, doublets, and k-ets (with or without beads) are $P(0)$, $P(1)$, $P(2)$, and $P(k)$, respectively. Therefore, the probability of multiplets is,

$$P(k \geq 2) = 1 - P(0) - P(1) = 1 - e^{-\lambda} - \lambda e^{-\lambda}$$

Supplementary Table 1 shows the probabilities of singlets, doublets, and triplets... based on the Poisson distribution. In this case, 0-et means an empty droplet, and these probabilities are among total droplets of which the number is usually unknown. Instead, what can be observed is the total number of droplets containing at least one cell (cell-droplets) (= $k \geq 1$), so the multiplet ratio, the fraction of multiplets among the cell-droplets can be expressed as

$$(multiplet\ ratio) = \frac{P(k \geq 2)}{P(k \geq 1)} = 1 - \frac{P(1)}{1 - P(0)} = 1 - \frac{\lambda e^{-\lambda}}{1 - e^{-\lambda}} \tag{1}$$

Also, the fraction of the singlet, doublet, triplet,... under the corresponding λ is shown in **Supplementary Table 2**. More than 80% was the singlet at around $\lambda \leq 0.4$. Indeed, the Chromium Controller (10x Genomics), which is one of the common microfluidics platforms for the droplet-based scRNA-seq, originally recommended the target cell number should be less than 10,000 cells, yielding 8% of multiplet among all cell-droplet, which corresponds to $\lambda = 0.166$. Loading cells exceeding this upper limit is regarded as "super-loading" ($\lambda > 0.166$) [5]. Indeed, at $\lambda = 0.5$, around 4% of multiplets are quartets or more, according to **Supplementary Table 2**. The relation of input cell numbers and λ in the Chromium Controller (10x Genomics) is further discussed in the next section.

k-et	$\lambda = 0.1$	$\lambda = 0.2$	$\lambda = 0.3$	$\lambda = 0.4$	$\lambda = 0.5$
0	0.9048374	0.8187308	0.7408182	0.6703200	0.6065307
1	0.0904837	0.1637462	0.2222455	0.2681280	0.3032653
2	0.0045242	0.0163746	0.0333368	0.0536256	0.0758163
3	0.0001508	0.0010916	0.0033337	0.0071501	0.0126361
4	0.0000038	0.0000546	0.0002500	0.0007150	0.0015795
5	0.0000001	0.0000022	0.0000150	0.0000572	0.0001580
6	0.0000000	0.0000001	0.0000008	0.0000038	0.0000132
7	0.0000000	0.0000000	0.0000000	0.0000002	0.0000009
8	0.0000000	0.0000000	0.0000000	0.0000000	0.0000001
9	0.0000000	0.0000000	0.0000000	0.0000000	0.0000000
10	0.0000000	0.0000000	0.0000000	0.0000000	0.0000000

Supplementary Table 1: The theoretical probability of droplets containing k cells

k-et	$\lambda = 0.1$	$\lambda = 0.2$	$\lambda = 0.3$	$\lambda = 0.4$	$\lambda = 0.5$
1	0.9508332	0.9033311	0.8574888	0.8132979	0.7707470
2	0.0475417	0.0903331	0.1286233	0.1626596	0.1926868
3	0.0015847	0.0060222	0.0128623	0.0216879	0.0321145
4	0.0000396	0.0003011	0.0009647	0.0021688	0.0040143
5	0.0000008	0.0000120	0.0000579	0.0001735	0.0004014
6	0.0000000	0.0000004	0.0000029	0.0000116	0.0000335
7	0.0000000	0.0000000	0.0000001	0.0000007	0.0000024
8	0.0000000	0.0000000	0.0000000	0.0000000	0.0000001
9	0.0000000	0.0000000	0.0000000	0.0000000	0.0000000
10	0.0000000	0.0000000	0.0000000	0.0000000	0.0000000

Supplementary Table 2: The theoretical ratio of the droplets containing k cells among all cell-droplets

2 λ in the Chromium Controller (10x Genomics)

Although the Poisson distribution is suitable to describe the probability of cell-droplets, the major drawback in practice is that we cannot see the total number of droplets, and, hence, cannot know the key parameter, λ, in a usual experimental setting. However, we can estimate it from an expected multiplet ratio. Here, we estimate λ in the Chromium Controller (10x Genomics). According to 10x Genomics, the multiplet rates with the Chromium Controller are shown in **Supplementary Figure 10A**. Here, $(mulitplet\ ratio) = 8 \times 10^{-6}N$, where N is the target cell number for analysis. Note that actual loading cell numbers are 165% of the target cell count to recover (from CG000315 Rev B Chromium Next GEM Single Cell 3' Reagent Kits v3.1 (Dual Index) USER GUIDE). Also, the doublet ratios in the HT kits (10x Genomics) are half that of the standard kit for the same target cell counts. These multiplet ratios can also be expressed with the Poisson distribution (**Equation** (**Eq.**) **1**). Therefore,

$$8 \times 10^{-6}N = 1 - \frac{\lambda e^{-\lambda}}{1 - e^{-\lambda}} \tag{2}$$

This relation between λ and the target cell counts is shown in **Supplementary Figure 10B**. Because this equation cannot be analytically solved for λ, a series of the estimated target cell number from λ by **Eq. 2**, were plotted, followed by approximation by linear regression (red line, **Supplementary Figure 10B**).

$$N \sim 56800\lambda + 570$$
$$\lambda \approx N/56800 - 0.01$$

The approximated λ for each target cell number in the standard kits is shown in **Supplementary Table 3**. The multiplet ratios, and hence, λ shall be half in the HT kits. In our two datasets (**Figure 2**) in this paper, we loaded cells to target 4,000 cells, corresponding to $\lambda = 0.060$. The CITE-seq dataset targeted 20,000 singlets + 5,000 multiplets (20%), corresponding $\lambda = 0.430$ by extrapolation. The NSCLC dataset targeted 40,000 cells with the HT kits, corresponding 20,000 cells ($\lambda = 0.160$) with the standard reagents in terms of the risk of multiplets. These λ, based on the device specification, are used as initial conditions for theoretical inference in **Supplementary Information Section ??**.

3 Probabilities of stealth multiplets under the multiple sample condition

Here, we examine the probabilities of the four categories of multiplets, p_{HS}, p_{PS}, p_{Mu}, and p_{Un} are the parts for the probability of homogeneous stealth, partial stealth, multilabelled, and unlabelled multiplets. The probabilities of empty droplets, singlets, and multiplets are expressed as $P(0)$, $P(1)$, and $P(k \geq 2)$, respectively. Therefore, the whole event is

$$\begin{aligned} P(0) + P(1) + P(k \geq 2) &= P(0) + P(1) + p_{\text{HS}} + p_{\text{PS}} + p_{\text{Mu}} + p_{\text{Un}} \\ &= 1 \end{aligned}$$

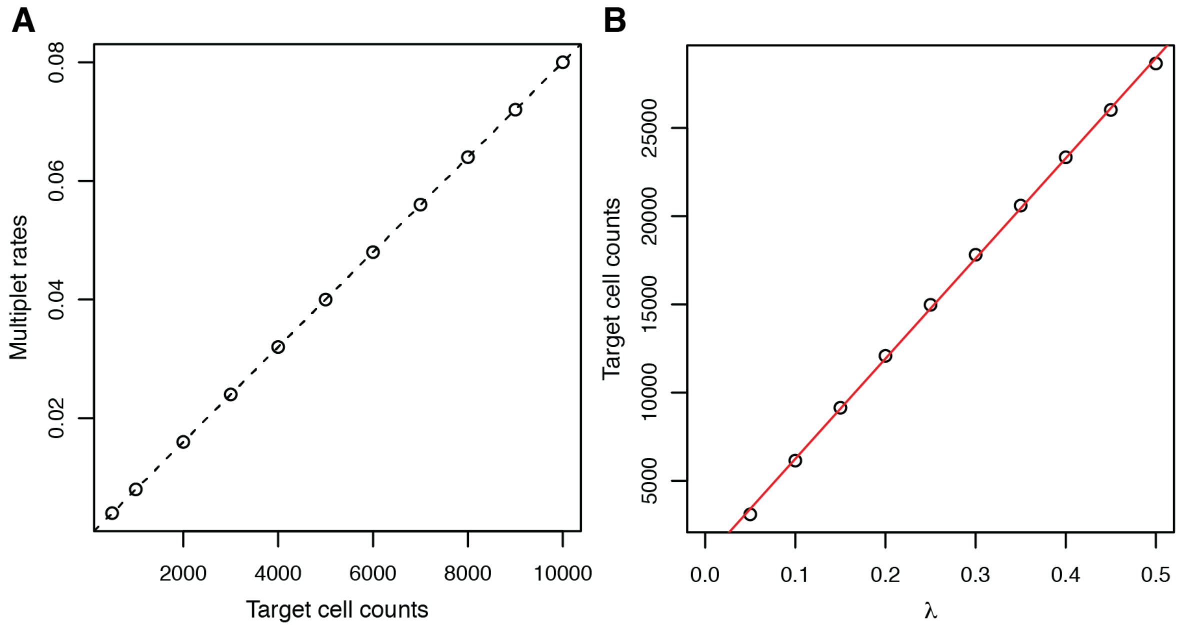

Supplementary Figure 10: (A) The relation between target cell counts and multiplet rates according to the Chromium Next GEM Single Cell 3' Reagent Kits v3.1 USER GUIDE (CG000315 Rev B). (B) The estimated relation between λ and target cell count. The points were plotted based on **Eq. 2**, and the red line was drawn by linear regression for approximation.

N	Multiplet ratio	λ
2000	0.016	0.025
4000	0.032	0.060
6000	0.048	0.096
8000	0.064	0.131
10000	0.080	0.166
12000	0.096	0.201
14000	0.112	0.236
16000	0.128	0.272
18000	0.144	0.307
20000	0.160	0.342
22000	0.176	0.377
24000	0.192	0.413

Supplementary Table 3: Estimated λ from the multiplet ratio in the Chromium Controller (10x Genomics)

Given s samples are labelled with a different barcode per sample under a labelling efficiency, a_i $(1 \leq i \leq s,\ 0 \leq a_i \leq 1)$, and s samples are pooled with a proportion of r_i $(1 \leq i \leq s,\ \sum_{i=1}^{s} r_i = 1)$, the overall average of labelling efficiencies, $\bar{a}$, is

$$\bar{a} = \sum_{i=1}^{s} r_i a_i$$

First, p_{HS} is expressed as the sum of homogeneous stealth multiplets of each sample as follows:

$$\begin{aligned} p_{\mathrm{HS}} &= \sum_{i=1}^{s} \sum_{k=2}^{\infty} \Big[P(k)(r_i a_i)^k \Big] \\ &= \sum_{i=1}^{s} \Big[e^{-\lambda} e^{r_i a_i \lambda} - r_i a_i P(1) - P(0) \Big] \\ &= e^{-\lambda} \Big(\sum_{i=1}^{s} e^{r_i a_i \lambda} - \bar{a}\lambda - s \Big) \end{aligned} \tag{3}$$

On the other hand, p_{Un} can be expressed as

$$\begin{aligned} p_{\mathrm{Un}} &= \sum_{k=2}^{\infty} P(k)(1-\bar{a})^k \\ &= e^{-\lambda}(e^{(1-\bar{a})\lambda} - (1-\bar{a})\lambda - 1) \end{aligned} \tag{4}$$

where $\bar{a}$ is the whole average of the labelling efficiency, because unlabelled multiplets are generated across samples, and we cannot distinguish the origin of cells consisting of unlabelled cell-droplets.

Next. we put the fraction of the homogeneous stealth multiplet, $f_{\mathrm{HS}}(k)$, and the unlabelled multiplets, $f_{\mathrm{Un}}(k)$, in each k-et cell-droplets.

$$\begin{aligned} f_{\mathrm{HS}}(k) &= \sum_{i=1}^{s} (r_i a_i)^k \qquad (k \geq 1) \\ f_{\mathrm{Un}}(k) &= (1-\bar{a})^k \qquad (k \geq 1) \end{aligned}$$

Here, we regard the labelled singlets and unlabelled singlets as special cases of homogeneous stealth multiplets and unlabelled multiplets, respectively, and extend the definition of $f_{\mathrm{HS}}(k)$, and $f_{\mathrm{Un}}(k)$ to $k = 1$. Note that these true singlets are not included in p_{HS} and p_{Un}. Then, the fraction of the partial stealth multiplet in k-et cell droplets, $f_{\mathrm{PS}}(k)$, can be described as the sum of the combinations of the fraction of homogeneous stealth j-et multiplets, $f_{\mathrm{HS}}(j)$, and unlabelled $(k-j)$-et multiplets, $f_{\mathrm{Un}}(k-j)$ $(1 \leq j \leq k-1)$. That is, $f_{\mathrm{PS}}(2)$ is a combination of $f_{\mathrm{HS}}(1)$ and unlabelled singlets, $f_{\mathrm{Un}}(1)$. $f_{\mathrm{PS}}(3)$ is the sum of the combination of $f_{\mathrm{HS}}(1)$ and $f_{\mathrm{Un}}(2)$, and $f_{\mathrm{HS}}(2)$ and $f_{\mathrm{Un}}(1)$. Similarly, $f_{\mathrm{PS}}(k)$ is the sum of the combination products of $f_{\mathrm{HS}}(1)$ and $f_{\mathrm{Un}}(k-1)$, $f_{\mathrm{HS}}(2)$ and $f_{\mathrm{Un}}(k-2)$, ..., and $f_{\mathrm{HS}}(k-1)$ and $f_{\mathrm{Un}}(1)$, and expressed as

$$\begin{aligned} f_{\mathrm{PS}}(k) &= \sum_{j=1}^{k-1} {}_kC_j f_{\mathrm{HS}}(j) f_{\mathrm{Un}}(k-j) \\ &= \sum_{j=1}^{k-1} {}_kC_j \Big(\sum_{i=1}^{s} (r_i a_i)^j \Big) \Big((1-\bar{a})^{k-j} \Big) \\ &= \sum_{i=1}^{s} \sum_{j=1}^{k-1} {}_kC_j (r_i a_i)^j (1-\bar{a})^{k-j} \\ &= \sum_{i=1}^{s} \Big[\sum_{j=0}^{k} {}_kC_j (r_i a_i)^j (1-\bar{a})^{k-j} - \underbrace{(r_i a_i)^k}_{j=k} - \underbrace{(1-\bar{a})^k}_{j=0} \Big] \\ &= \sum_{i=1}^{s} (r_i a_i + 1 - \bar{a})^k - f_{\mathrm{HS}}(k) - s f_{\mathrm{Un}}(k) \end{aligned}$$

From this equation, the probability of both types of stealth multiplets, $p_{\mathrm{HS}} + p_{\mathrm{PS}}$ is expressed as follows:

$$
\begin{aligned}
p_{\mathrm{HS}} + p_{\mathrm{PS}} &= p_{\mathrm{HS}} + \sum_{k=2}^{\infty} P(k) f_{\mathrm{PS}}(k) \\
&= p_{\mathrm{HS}} + \sum_{k=2}^{\infty} P(k) \Big[\sum_{i=1}^{s} (r_i a_i + 1 - \bar{a})^k - f_{\mathrm{HS}}(k) - s f_{\mathrm{Un}}(k) \Big] \\
&= \sum_{i=1}^{s} \sum_{k=2}^{\infty} P(k) (r_i a_i + 1 - \bar{a})^k - s p_{\mathrm{Un}} \\
&= \sum_{i=1}^{s} \left[e^{-\lambda} e^{\lambda (r_i a_i + 1 - \bar{a})} - (r_i a_i + 1 - \bar{a}) \lambda e^{-\lambda} - e^{-\lambda} \right] - s p_{\mathrm{Un}} \\
&= e^{-\bar{a}\lambda} \sum_{i=1}^{s} e^{r_i a_i \lambda} - \bar{a}\lambda e^{-\lambda} - s(1-\bar{a})\lambda e^{-\lambda} - s e^{-\lambda} \\
&\quad - s e^{-\lambda} (e^{(1-\bar{a})\lambda} - (1-\bar{a})\lambda - 1) \\
&= e^{-\bar{a}\lambda} \sum_{i=1}^{s} e^{r_i a_i \lambda} - \bar{a}\lambda e^{-\lambda} - s e^{-\bar{a}\lambda}
\end{aligned}
\tag{5}
$$

From the **Eq. 3, 4, and 5**, we can express the probability of the other categories of multiplets. First, the probability of the partial stealth multiplet p_{PS} is

$$
\begin{aligned}
p_{\mathrm{PS}} &= (p_{\mathrm{HS}} + p_{\mathrm{PS}}) - p_{\mathrm{HS}} \\
&= e^{-\bar{a}\lambda} \sum_{i=1}^{s} e^{r_i a_i \lambda} - \bar{a}\lambda e^{-\lambda} - s e^{-\bar{a}\lambda} - e^{-\lambda} \Big(\sum_{i=1}^{s} e^{r_i a_i \lambda} - \bar{a}\lambda - s \Big) \\
&= (e^{-\bar{a}\lambda} - e^{-\lambda}) \Big(\sum_{i=1}^{s} e^{r_i a_i \lambda} - s \Big)
\end{aligned}
\tag{6}
$$

Then, the probability of the multilabelled droplet p_{Mu} can be expressed as the complement of the other three types of multiplets ($p_{\mathrm{Mu}} = 1 - P(0) - P(1) - (p_{\mathrm{HS}} + p_{\mathrm{PS}} + p_{\mathrm{Un}})$). From **Eq. 4 and 5**, the sum of p_{HS}, p_{PS}, and p_{Un} is expressed as

$$
\begin{aligned}
p_{\mathrm{HS}} + p_{\mathrm{PS}} + p_{\mathrm{Un}} &= e^{-\bar{a}\lambda} \sum_{i=1}^{s} e^{r_i a_i \lambda} - \bar{a}\lambda e^{-\lambda} - s e^{-\bar{a}\lambda} + e^{-\lambda} (e^{(1-\bar{a})\lambda} - (1-\bar{a})\lambda - 1) \\
&= e^{-\bar{a}\lambda} \Big(\sum_{i=1}^{s} e^{r_i a_i \lambda} - s + 1 \Big) - e^{-\lambda} - \lambda e^{-\lambda} \\
&= e^{-\bar{a}\lambda} \Big(\sum_{i=1}^{s} e^{r_i a_i \lambda} - s + 1 \Big) - P(0) - P(1)
\end{aligned}
$$

Therefore, the probability of the multilabelled multiplet is

$$
\begin{aligned}
p_{\mathrm{Mu}} &= 1 - P(0) - P(1) - (p_{\mathrm{HS}} + p_{\mathrm{PS}} + p_{\mathrm{Un}}) \\
&= 1 - P(0) - P(1) - \bigg(e^{-\bar{a}\lambda} \Big(\sum_{i=1}^{s} e^{r_i a_i \lambda} - s + 1 \Big) - P(0) - P(1) \bigg) \\
&= 1 - e^{-\bar{a}\lambda} \Big(\sum_{i=1}^{s} e^{r_i a_i \lambda} - s + 1 \Big)
\end{aligned}
\tag{7}
$$

Lastly, when s samples are multiplexed, the overall *TSR* is

$$\begin{aligned} TSR &= \frac{P(1)\sum_{i=1}^{s} r_i a_i}{P(1)\sum_{i=1}^{s} r_i a_i + p_{\text{HS}} + p_{\text{PS}}} \\ &= \frac{\bar{a}\lambda e^{-\lambda}}{\bar{a}\lambda e^{-\lambda} + e^{-\bar{a}\lambda}\sum_{i=1}^{s} e^{r_i a_i \lambda} - \bar{a}\lambda e^{-\lambda} - s e^{-\bar{a}\lambda}} \\ &= \frac{\bar{a}\lambda e^{-\lambda(1-\bar{a})}}{\sum_{i=1}^{s} e^{r_i a_i \lambda} - s} \end{aligned} \tag{8}$$

4 Assuming equal labelling efficiency and pooling proportions in all samples

To facilitate the exploration of the behaviour of the probability of the multiplets in **Figures 1C-1F** and **Supplementary Figure 1**, we further assume all samples are labelled evenly and pooled with the same proportion. in this case, $a_i = \bar{a}$ and $r_i = 1/s$ for given i, and thus,

$$r_i a_i = \frac{\bar{a}}{s} \tag{9}$$

Under this condition, the probability of each category (**Eq. 3, 6, and** 7) can be expressed as,

$$\begin{aligned} p_{\text{HS}} &= s e^{-\lambda}(e^{\frac{\bar{a}\lambda}{s}} - 1) - \bar{a}\lambda e^{-\lambda} \\ p_{\text{PS}} &= s(e^{-\bar{a}\lambda} - e^{-\lambda})(e^{\frac{\bar{a}\lambda}{s}} - 1) \\ p_{\text{Mu}} &= 1 - e^{-\bar{a}\lambda}\big(s(e^{\frac{\bar{a}\lambda}{s}} - 1) + 1\big) \end{aligned} \tag{10}$$

From these equations, together with the probability of the unlabelled multiplet (**Eq. 4**), we can explore their behaviour by considering only $\bar{a}$ and λ. Similarly, from **Eq. 8**, the *TSR* under this condition is

$$TSR = \frac{\bar{a}\lambda e^{-\lambda(1-\bar{a})}}{s(e^{\frac{\bar{a}\lambda}{s}} - 1)} \tag{11}$$

This assumption averages the products of labelling efficiency and a proportion of each sample in the whole pool. In other words, this assumption averages the contribution of each sample to the entire labelling performance. Therefore, examining deviations, if any, from what is estimated from heterogeneous sample labelling is useful. To represent heterogeneous pooling, first, we test the situation that one of the pooled samples is ill-labelled whereas others have equal efficiency, and the overall average labelling efficiency $\bar{a}$ is constant as shown on top of the graphs (**Supplementary Figures 11A and 11B**). For example, when $\bar{a} = 0.8$ and the number of samples is 5, if the worst labelling efficiency is 0.6, the labelling efficiency of the rest of the 4 samples is 0.85. **Supplementary Figure 11A** shows four conditions of the parameter when the number of samples is 5 or 2, and $\bar{a} = 0.8$ or $\bar{a} = 0.5$. The red lines show the *TSR* when the assumption **Eq. 9** holds (under $\lambda = 0.5$). In all conditions, the deviation is greater when the λ is larger and when the number of samples is smaller, though the gaps are small except under $\bar{a} = 0.5$ and the number of samples is 2. Indeed, comparing the multiple numbers of samples under $\lambda = 0.3$, we see the deviation is substantial when two samples are multiplexed but not when five samples (**Supplementary Figure 11B**). Therefore, when the number of samples is at least 5 or $\bar{a} \geq 0.8$, the averaged labelling efficiency $\bar{a}$ is a good approximation.

On the other hand, when the proportion of the pooled samples is different, even though the labelling efficiency is the same across all samples, the deviation can be greater (**Supplementary Figure 11C**). This is probably because the number of the homogeneous stealth multiplet is greater, which makes it difficult to remove the multiplet as a multilabelled multiplet. However, it is an extreme case that the proportion of one sample is nearly 1, because it means a single sample mostly occupies the whole sample. Indeed, if one sample is twice as many as the rest (0.4 when $s = 5$), the deviation is 1-2%, and the averaged labelling efficiency $\bar{a}$ is still a good approximation. This analysis also confirms that the highest *TSR* can be achieved in theory when the number of cells in each sample is equal.

Overall, these results showed the effect of ill-labelled samples is relatively small within a usual experimental design and technical errors, and $\bar{a}$ can be used to approximate the overall *TSR* as long as an experiment intends to pool the same number of cells from each sample, and the estimated $\bar{a}$ or the number of samples multiplexed is high enough.

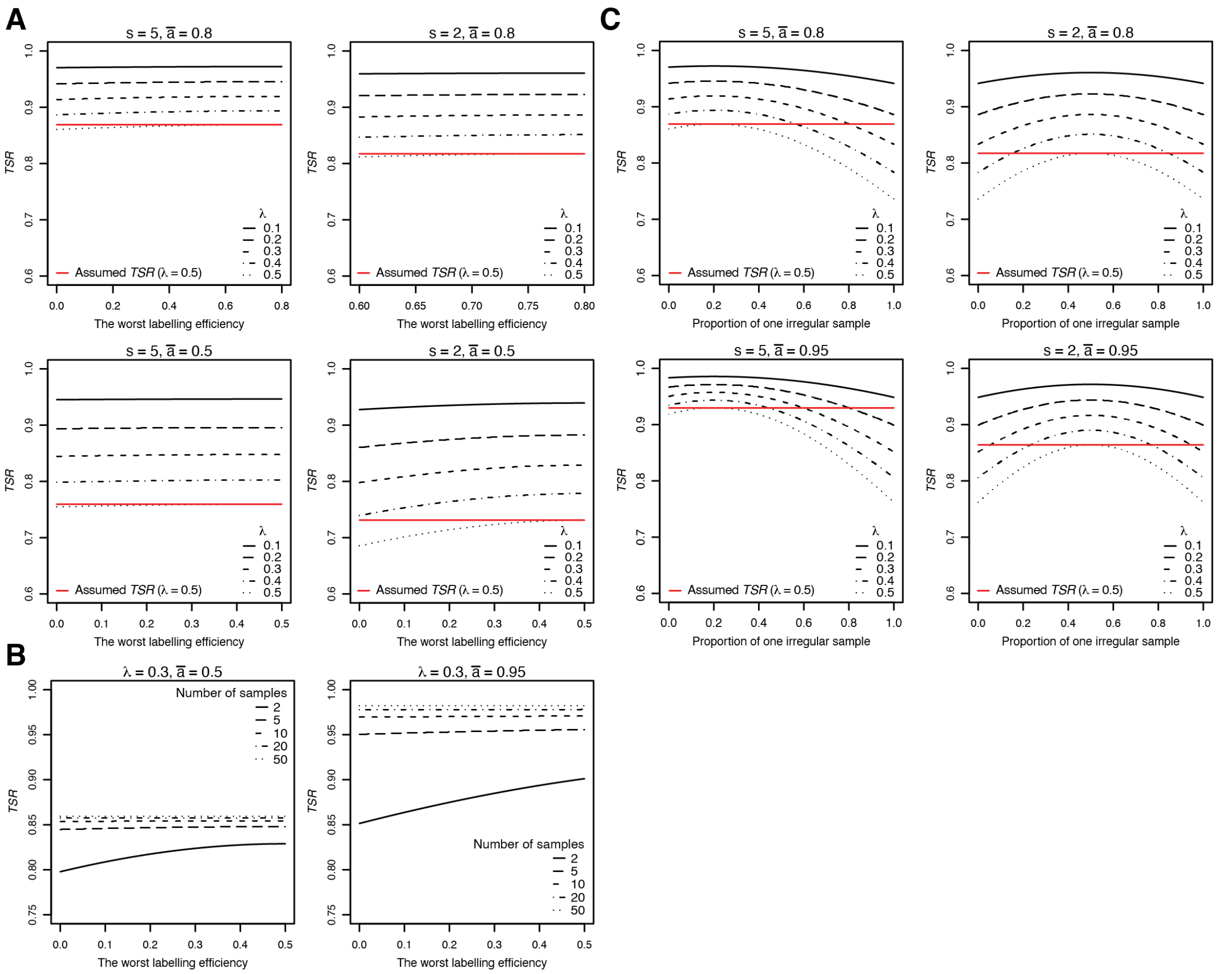

Supplementary Figure 11: (A) *TSR* is plotted against the worst labelling efficiency among the multiplexed samples under various λ. In this scenario, one sample's labelling efficiency is worse than the others, and the rest of the samples have the same labelling efficiency, and $\bar{a}$, the average labelling efficiency across all samples, is constant. The fixed conditions, the number of samples, s, and $\bar{a}$, are shown on top of each graph. (top left) $s = 5$ and $\bar{a} = 0.8$, (top right) $s = 2$ and $\bar{a} = 0.8$, (bottom left) $s = 5$ and $\bar{a} = 0.5$, (bottom right) $s = 2$ and $\bar{a} = 0.5$. (B) Similar to (A), *TSR* is plotted against the worst labelling efficiency under different sample numbers. The fixed conditions, λ and $\bar{a}$ are shown on top of each graph. (left) $\lambda = 0.3$ and $\bar{a} = 0.5$, (right) $\lambda = 0.3$ and $\bar{a} = 0.95$. (C) *TSR* is plotted against the proportion of one sample in the sample pool under various λ. In this scenario, one sample's proportion is different from the others, and the rest of the samples were pooled with the same proportions, and the whole sum of the proportions equals 1. The fixed conditions, the number of samples, s, and $\bar{a}$, are shown on top of each graph. (top left) $s = 5$ and $\bar{a} = 0.8$, (top right) $s = 2$ and $\bar{a} = 0.8$, (bottom left) $s = 5$ and $\bar{a} = 0.95$, (bottom right) $s = 2$ and $\bar{a} = 0.95$.

5 Estimating λ and a labelling efficiency from demultiplexing results

Because the total droplet count is unknown, the only clues to estimate the parameters, λ and the average labelling efficiency, $\bar{a}$, are the ratio of observed cell numbers, particularly that of monolabelled to unlabelled cell-droplets, and the number of observed multilabelled droplets. In this study, first, we infer the λ of each experiment by using the cell counts of the "ground truth" data (transcriptome-based demultiplexing in the mouse datasets, and oligonucleotide-barcode-based demultiplexing in the human datasets).

Given that the labelling efficiency is close to 1 (or practically 1 in the transcriptome-based demultiplexing), the average labelling efficiency $\bar{a}$ can be approximated by

$$\bar{a} \approx \frac{N_{monolabelled}}{N_{monolabelled} + N_{unlabelled}} \tag{12}$$

where $N_{monolabelled}$ and $N_{unlabelled}$ were the numbers of monolabelled and unlabelled droplets from the demultiplexing results, respectively. On the other hand, from **Eq. 10** the ratio of the observed multilabelled droplets among the total cell-droplets can be described as

$$\frac{N_{multilabelled}}{N_{total}} = \frac{p_{\text{Mu}}}{1 - P(0)} = \frac{1 - e^{-\bar{a}\lambda}\big(s(e^{\frac{\bar{a}\lambda}{s}} - 1) + 1\big)}{1 - e^{-\lambda}}$$

where $N_{multilabelled}$ and N_{total} were the numbers of multilabelled droplets and total cell-droplets from the demultiplexing results, respectively. Here, we also adopt the assumption **Eq. 9**. By substituting the ratio from **Eq. 12** for $\bar{a}$, we can numerically solve for λ. In this study, we used the Newton-Raphson method with the multiroot function of rootSolve package (version 1.8.2.3) in R (version 4.1.2). The initial condition for λ is theoretically estimated λ from the target number of the cells (see **Supplementary Information Section ??** above). The summary of the estimation is shown in **Supplementary Table 4**.

	Observed results				Target		Estimation	
Dataset	Mono	Multi	Unlab	Multi(%)	Multiplet(%)	λ	λ	$\bar{a}$
Two-sample	1583	62	0	3.77	3.2	0.060	0.151	1.000
Three-sample	2646	51	0	1.89	3.2	0.060	0.057	1.000
CITE	14266	2578	72	15.24	20.0	0.430	0.369	0.995
NSCLC	24805	7864	0	24.07	16.0	0.342	0.608	1.000

Supplementary Table 4: λ of each experiment is estimated from the observed monolabelled, multilabelled, and unlabelled droplet counts of the reference demultiplexing results shown in **Figures 2, 3, and 4**. The expected multiplet ratio and λ from the device specification are shown in the middle ("Target"), and the estimated values from the droplet counts are on the right. Mono, monolabelled droplet, Multi, multilabelled droplet, Unlab, Unlabelled droplet.

When there are no unlabelled droplets, $\bar{a}$ is 1, and the risk of the partial stealth multiplet cannot be foreseen in theory. Also, even in the case of the CITE-seq dataset, the expected proportion of the partial stealth multiplet is $< 0.2\%$ according to **Eq. 10**. Therefore, the oligonucleotide-barcode-based demultiplexing results in the CITE-seq and NSCLC datasets can be regarded as references about the origin of cells. Moreover, from these inferred λ, we can further estimate the theoretical ratio of the four categories of multiplets in the demultiplexing results tested (oligonucleotide-barcode-based demultiplexing by GMM-demux in the mouse datasets, and SNP-based demultiplexing by demuxalot in the human datasets). To this end, we first estimate $\bar{a}$ in each demultiplexing result by utilising the ratio of monolabelled to unlabelled droplets. Unlike the reference demultiplexing results, $N_{unlabelled} \neq 0$ in these results, and the ratio can be calculated. From **Eq. 4** and **10**, also by assuming that the labelling efficiencies of individual samples are equal, and the equal proportions of cells are pooled (**Eq. 9**), the probabilities of monolabelled and unlabelled cell-droplets are

$$P_{monolabelled} = \bar{a}P(1) + p_{\text{HS}} + p_{\text{PS}} = se^{-\bar{a}\lambda}(e^{\frac{\bar{a}\lambda}{s}} - 1)$$
$$P_{unlabelled} = (1 - \bar{a})P(1) + p_{\text{Un}} = e^{-\bar{a}\lambda} - e^{-\lambda}$$

Therefore, the ratio of monolabelled to unlabelled droplets is

$$P_{monolabelled}/P_{unlabelled} = \frac{s(e^{\frac{\bar{a}\lambda}{s}} - 1)}{1 - e^{-\lambda(1-\bar{a})}} \tag{13}$$

This equation can be solved for $\bar{a}$ numerically, and then we can estimate the ratio of the four categories of multiplets with **Eq. 10** and **Eq. 4**. The results are shown in **Supplementary Table 5**.

	Observed results					Parameter		Estimation				
Dataset	Mono	Multi	Unlab	PS(%)	Mu(%)	λ	$\bar{a}$	HS(%)	PS(%)	Mu(%)	Un(%)	*TSR*
Two-sample	1211	31	403	2.80	1.88	0.151	0.741	1.96	2.81	2.11	0.47	0.935
Three-sample	2596	82	19	0.07	3.04	0.057	0.993	0.91	0.04	1.86	0.00	0.990
CITE	14319	1877	680	2.37	4.68	0.369	0.941	1.71	1.76	13.79	0.05	0.957
NSCLC	28825	3756	88	11.65	10.22	0.608	0.996	3.22	0.17	23.94	0.00	0.955

Supplementary Table 5: The observed monolabelled, multilabelled, and unlabelled droplet counts are from the oligonucleotide-barcode-based (in Two-sample and Three-sample datasets) or SNP-based (CITE and NSCLC datasets) demultiplexing results (**Figures 2, 3, and 4**). The observed proportion of the partial stealth multiplet (PS), and the multilabelled multiplet (Mu) is based on the data shown in **Figures 2D and 2H**, **Supplementary Figure 2E**, **Supplementary Figures 4B-4D**, and **Figures 3C and 4C**. λ are from **Supplementary Table 4**. $\bar{a}$ are estimated from the observed droplet counts and λ by solving **Eq. 13**, and the percentages of the four categories of multiplets among the whole cell-droplet and *TSR* are estimated with **Eq. 4, 10, and 11** . Mono, monolabelled droplet, Multi, multilabelled droplet, Unlab, Unlabelled droplet, HS, Homogeneous stealth multiplet. Un, Unlabelled multiplet

According to the estimation, the expected numbers of partial stealth multiplets are 46 and 1 in the two-sample and three-sample datasets, respectively. They are consistent with the GMM-demux demultiplexing results, which were 39 and 2, respectively (**Figures 2D and 2H**, **Supplementary Figure 2E**, and **Supplementary Figures 4B-4D**). Note that the partial stealth multiplet was estimated to be the most prevalent type among the four categories in the poorly labelled "two-sample" dataset. On the contrary, there is a discrepancy between the estimation and the SNP-based demultiplexing results, which is probably due to the under-detection of multilabelled multiplets.

References

1. Zheng, G. X. Y. *et al.* Massively parallel digital transcriptional profiling of single cells. en. *Nature Communications* **8,** 14049. ISSN: 2041-1723. https://www.nature.com/articles/ncomms14049 (Jan. 2017).

2. McGinnis, C. S., Murrow, L. M. & Gartner, Z. J. DoubletFinder: Doublet Detection in Single-Cell RNA Sequencing Data Using Artificial Nearest Neighbors. en. *Cell Systems* **8,** 329–337.e4. ISSN: 24054712. https://linkinghub.elsevier.com/retrieve/pii/%20S2405471219300730 (Apr. 2019).

3. Bloom, J. D. Estimating the frequency of multiplets in single-cell RNA sequencing from cell-mixing experiments. en. *PeerJ* **6,** e5578. ISSN: 2167-8359. https://peerj.com/articles/5578 (Sept. 2018).

4. Xin, H. *et al.* GMM-Demux: sample demultiplexing, multiplet detection, experiment planning, and novel cell-type verification in single cell sequencing. en. *Genome Biology* **21,** 188. ISSN: 1474-760X. https://genomebiology.biomedcentral.com/articles/10.1186/%20s13059-020-02084-2 (Dec. 2020).

5. Stoeckius, M. *et al.* Cell Hashing with barcoded antibodies enables multiplexing and doublet detection for single cell genomics. *Genome Biology* **19,** 224. ISSN: 1474-760X. https://doi.org/10.1186/s13059-018-1603-1 (Dec. 2018).
